# Potential role of polycyclic aromatic hydrocarbons as mediators of cardiovascular effects from combustion particles

**DOI:** 10.1186/s12940-019-0514-2

**Published:** 2019-08-22

**Authors:** Jørn A. Holme, Bendik C. Brinchmann, Magne Refsnes, Marit Låg, Johan Øvrevik

**Affiliations:** 1 Department of Air Pollution and Noise, Division of Infection Control and Environmental Health, Norwegian Institute of Public Health, PO Box 222, Skøyen, N-0213 Oslo, Norway; 2 Department of Biosciences, Faculty of Mathematics and Natural Sciences, University of Oslo, Oslo, Norway

**Keywords:** Air pollution, Combustion particles, Polycyclic aromatic hydrocarbons, Cardiovascular disease, Atherosclerosis

## Abstract

Air pollution is the most important environmental risk factor for disease and premature death, and exposure to combustion particles from vehicles is a major contributor. Human epidemiological studies combined with experimental studies strongly suggest that exposure to combustion particles may enhance the risk of cardiovascular disease (CVD), including atherosclerosis, hypertension, thrombosis and myocardial infarction.

In this review we hypothesize that adhered organic chemicals like polycyclic aromatic hydrocarbons (PAHs), contribute to development or exacerbation of CVD from combustion particles exposure. We summarize present knowledge from existing human epidemiological and clinical studies as well as experimental studies in animals and relevant in vitro studies. The available evidence suggests that organic compounds attached to these particles are significant triggers of CVD. Furthermore, their effects seem to be mediated at least in part by the aryl hydrocarbon receptor (AhR). The mechanisms include AhR-induced changes in gene expression as well as formation of reactive oxygen species (ROS) and/or reactive electrophilic metabolites. This is in accordance with a role of PAHs, as they seem to be the major chemical group on combustion particles, which bind AhR and/or is metabolically activated by CYP-enzymes. In some experimental models however, it seems as PAHs may induce an inflammatory atherosclerotic plaque phenotype irrespective of DNA- and/or AhR-ligand binding properties. Thus, various components and several signalling mechanisms/pathways are likely involved in CVD induced by combustion particles.

We still need to expand our knowledge about the role of PAHs in CVD and in particular the relative importance of the different PAH species. This warrants further studies as enhanced knowledge on this issue may amend risk assessment of CVD caused by combustion particles and selection of efficient measures to reduce the health effects of particular matters (PM).

## Background

According to the World Health Organization (WHO) air pollution is the preponderant environmental risk factor, being responsible for about one in every nine deaths globally [1]. Exposure to particular matter with an aerodynamic diameter of 2.5 μm and less (PM_2.5_) has been found to have vascular effects leading to ischemia, myocardial infarction, stroke and other cardiovascular diseases (CVD) [2–4]. Cardiovascular health consequences of air pollution are generally equal to or exceed those due to pulmonary diseases [3, 5]. As is the case for lung cancer, it is no apparent threshold for adverse cardiovascular effects due to PM_2.5_ in the dose range humans are exposed [6]. The aim of this review was to highlight the hazard potential of polycyclic aromatic hydrocarbons (PAHs) as mediators of PM-induced CVD, as this has received limited attention by particle toxicologists.

### Particulate matter and polycyclic aromatic hydrocarbons in ambient air

A number of factors affects PM toxicity, including size, shape, structure, surface reactivity, bio-persistence and presence of soluble components (Table 1) [7, 8]. There are large regional differences in composition of PM depending on sources [9]. Toxicological studies have identified several transition metals, organic carbon species, semi-quinones, and endotoxins as specific PM-related components with potential to induce oxidative stress and inflammation [3]. Combustion engines, in particular diesel engines are major contributors to PM_2.5_ in urban environments. Thus, combustion particles such as diesel exhaust particles (DEP) are frequently used to explore mechanisms of PM-induced CVD [7, 10–12]. Combustion particles consist of carbon cores in the ultrafine PM size-range (< 100 nm) with complex mixtures of organic chemicals adhered to the surface [13, 14]. Composition and amount of organic chemicals present in DEP vary, dependent on fuel burned, temperature, engine load, drive-cycles and type of combustion technology. Average levels of organic chemicals in DEP generally range from 20 to 40% of total mass, but may reach as much as 90% [15, 16]. PAHs are the most well-known of these chemicals [17]. Other known chemical groups include n-alkanes, hopanes and steranes [18].

**Table 1:**
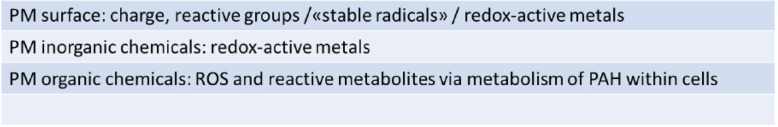
Combustion particle properties linked to redox activity

PAHs are produced by incomplete combustion of organic materials such as coal and fossil fuels, cigarette smoking and various industrial activities [19]. The major sources for the global total atmospheric emission of PAH16 have been estimated to be residential/commercial biomass burning (60.5%), open-field biomass burning (agricultural waste burning, deforestation, wildfires (13.6%), and petroleum consumption by motor vehicles (12.8%) [20]. The majority of airborne PAHs with low vapor pressure is adsorbed to PM [17, 21]. PAHs containing five or more aromatic rings are mainly found bound to PM, while PAHs containing four or less aromatic rings seem predominately to occur in the gas phase. However, three and four-ring PAHs such as phenanthrene and pyrene are so abundant in outdoor air, that they also tend to be the most abundant PAHs bound to DEP and other combustion particles [17]. As temperature and vapor pressure is connected, a considerably larger proportion of airborne PAHs will be bound to PM during winter, while a relatively larger fraction will be in the gas phase during summer. In line with this, winter PM_2.5_ from Milan (Italy) was found to contain 10-fold higher PAH content (% of PM mass) compared to summer PM_2.5_ [22].

### Possible mechanisms linking PM to CVD

WHO has estimated that approximately 75% of deaths attributable to ambient air pollution are due to stroke or ischemic heart disease [1]. PM_2.5_ exposure is associated with endothelial dysfunction in CVD–risk groups [23, 24], but recent findings indicate that environmental exposure to PM_2.5_ may cause endothelial injury even in young healthy adults [25]. Furthermore, it has been suggested that air pollution may cause hypertension, and increase in long-term PM_2.5_ exposure as low as 3 μg/m^3^ has been associated with vascular dysfunction [26, 27]. Double-blinded cross-over exposures have also revealed that diesel exhaust increases systolic blood pressure in healthy participants [28].

**Fig. 1:**
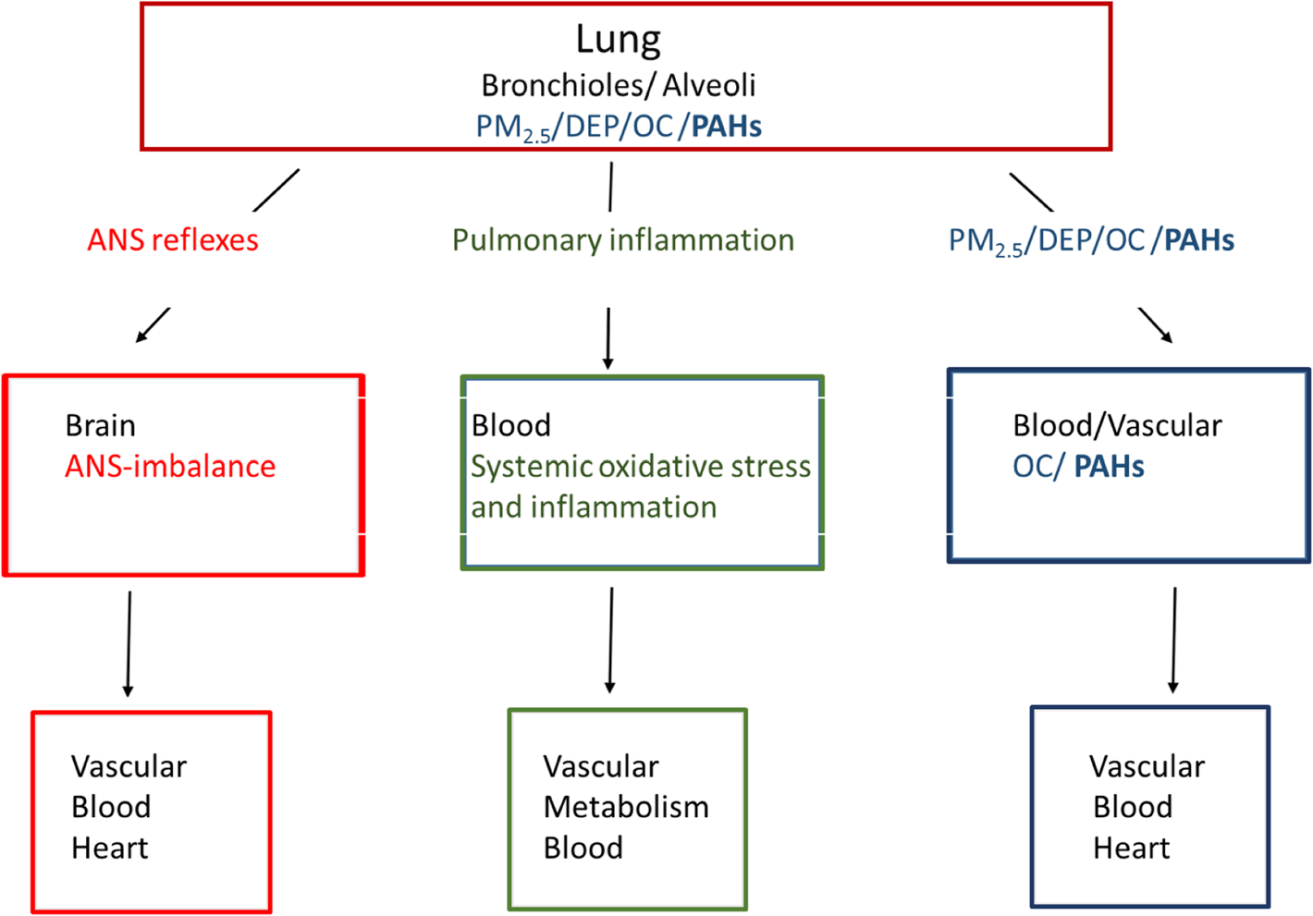
Possible mechanisms linking PM2.5/ DEP/ OC/ PAH with CVD. Three general lines of causality are suggested: i) Distortion of autonomic nerve endings in the lungs causing loss of vascular control reflexes via the autonomic nervous system (ANS; red), ii) Pulmonary inflammation and “systemic spill over” (green) and iii) direct effects of organic chemicals (OC) and polycyclic aromatic hydrocarbons (PAHs), affecting blood/vascular system directly (blue). Possible links include: oxidative stress, inflammation, vasoconstriction, endothelial dysfunction, coagulation, thrombosis, heart rate, heart rate variability (HRV), redox imbalance, impaired high density lipoproteins (HDL)-function as well as effects during embryonic development - via reactive metabolites, reactive oxygen species (ROS), aryl hydrocarbon receptor (AhR)-genomic and/or non-genomic pathways including [Ca^2+^]_I_ and G protein-coupled receptors (GPCRs). Partly modified from [3]

Combustion particles may contribute to development of CVD via several mechanisms (Fig. 1 and Table 2). Exposure of pulmonary macrophages and epithelial cells may cause oxidative stress, further triggering release of pro-inflammatory mediators into the circulation. These mediators have potential to harm endothelial cells and cause systemic effects [25, 29]. PM_2.5_/DEP may affect platelets and coagulation, increasing the risk of vascular clotting [30–32]. It has also been suggested that inhaled diesel exhaust may trigger receptors in the autonomic nervous system of the respiratory tract and thus affect cardiac control [33, 34]. In addition, constituents of PM_2.5_/DEP may have more direct cardiovascular effects [11, 35, 36]. Recently, inhaled gold nanoparticles were found to accumulate at sites of vascular inflammation in mice and humans [37]. However, only a small amount of gold nano-particles (less than 0.3%) reach the circulation [38]. By contrast, it has been shown that when combustion particles deposit in the alveolar region the majority of their available PAH-load may rapidly detach from the particles, and is transferred across the epithelial barrier and diffuses into the bloodstream in an un-metabolized state [17, 39, 40].

**Table 2:**
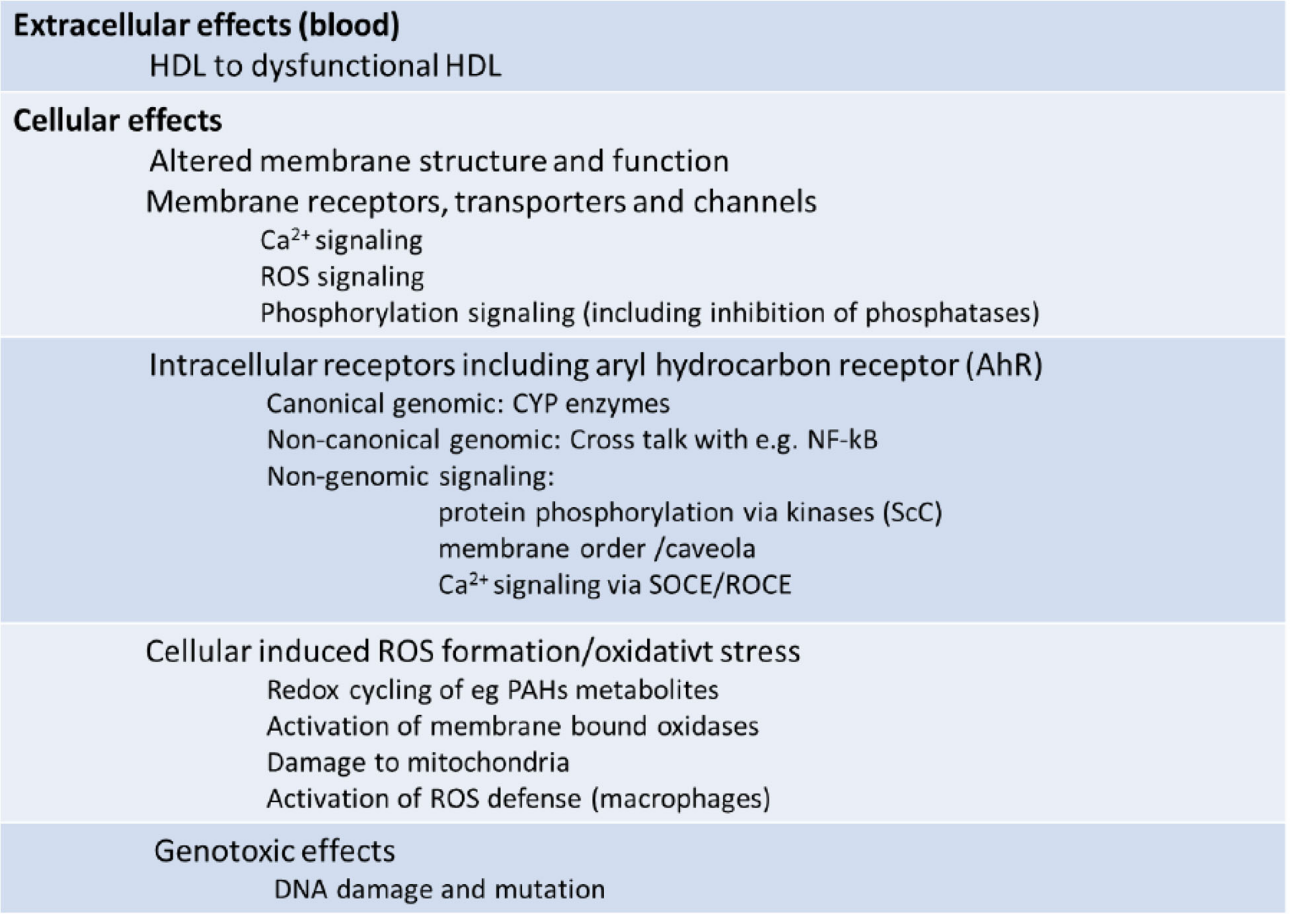
Initial molecular effects of combustion particle/PAH-Parent compound, reactive oxygen species (ROS) and electrophilic metabolites

Due to the complex composition of PM_2.5_, there is no single causative chemical, chemical group or component behind the various cardiovascular effects [3, 41, 42]. However, even though particle cores sometimes may be involved, biologic effects of combustion particles seem largely dependent on organic chemicals. Notably, animal studies have shown that DEP denuded of organic chemicals lost their potential to induce atherosclerosis [43]. Furthermore, experimental studies in vitro have illustrated that some effects of PM_2.5_/DEP relevant for CVD, are linked to extractable chemicals from these particles [44–48]. Thus, as PM_2.5_/DEP contains substantial amounts of organic chemicals, their vascular effects may presumably be linked to these chemicals [11, 14, 35, 37].

### Inflammation and atherosclerosis

Atherosclerosis may lead to myocardial infarction, cerebrovascular and peripheral vascular disease, making it the major cause of deaths due to CVD [49, 50]. It is an inflammatory disorder of the arteries, initiated by dysfunction of endothelial cells, which precede the histo-pathological changes. The process involves oxidative stress and leads to increased levels of local inflammatory mediators including cytokines, chemokines and adhesion molecules that lead to extravasation of monocytes. These monocytes accumulate oxidized low-density lipoproteins (oxLDL) and develop into foam cells and deteriorate, leading to atheroma. Several mediators among others matrix metalloproteinases (MMPs) destabilize atherosclerotic plaques ultimately causing rupture and thus infarction [51].

Inflammation in endothelial cells and/or the lung is considered a central link between ambient PM-exposure and CVD [52]. Inflammatory reactions may be directly caused by PM-induced chemokine/cytokine release as well as indirectly through PM-induced cytotoxicity [53, 54]. Oxidative stress is central in both processes [54–56]. Reactive oxygen species (ROS) can be generated directly by particles and particle components or more indirectly through various metabolic and inflammatory processes (Tables 1 and 2) [57, 58].

After exposing healthy men to DEP, Törnqvist and co-workers observed impairment of endothelium-dependent vasodilatation suggested to be due to early systemic oxidative stress [59]. Animal experiments have shown that DEP exposure increases size and complexity of lesions in atherosclerotic mice [60]. In an Apo E^−/−^ mice model, DEP caused marked effects on buildup of plaques in arterial walls, while DEP denuded of organic chemicals was without effect [43], indeed supporting an important role of these chemicals in atherosclerotic effects of DEP. That DEP may aggravate development and progression of atherosclerosis is further supported by in vitro studies. In a co-culture model, wood smoke particles and DEP increased adhesion of monocytes to endothelial cells [61], which is often linked to enhanced migration of inflammatory cells from the bloodstream. DEP has been shown to impair endothelial function [62, 63], increase formation of lipid-loaded foam cells from macrophages [64], and trigger inflammatory reactions in endothelial cells [48].

### Aryl hydrocarbon receptor

The aryl hydrocarbon receptor (AhR), plays a central role in regulating toxicity of PAHs and other environmental pollutants such as dioxins and co-planar polychlorinated biphenyls [65, 66]. In its classical mode of action, ligand-activated AhR dimerizes with the AhR nuclear translocator (ARNT) and binds to so-called xenobiotic response elements (XREs) in promotor regions of target genes such as cytochrome P450 (CYP) enzymes CYP1A1/CYP1B1 (Table 2). Metabolism of PAH from DEP by various CYP-enzymes may form ROS and reactive electrophilic metabolites with potential to trigger inflammation [67, 68]. Furthermore, it has now become clear that a number of pro-inflammatory genes are directly regulated by the AhR [69–71], and at least some of these such as interleukin (IL)-1β and IL-8 (CXCL8) contain xenobiotic response elements (XREs) in their promotor region [72, 73]. AhR may also mediate inflammatory signals via non-classical pathways; this includes cross-talk with the nuclear factor-κB (NF-κB) family of transcription factors as well as other transcription factors and signaling molecules, independent of ARNT activation [74–76]. In addition to its transcriptional role, AhR is also believed to mediate toxic effects via non-genomic signals including increases in intracellular concentration of calcium [Ca^2+^]_*i*_ [77, 78].

AhR is important for cellular functions. Increasing evidence suggests that AhR plays a central role in development and maintenance of the cardiovascular system, and that xenobiotics may affect homeostasis and trigger CVD-pathogenesis by modulating biological responses of critical cell types through activation of AhR [79–84]. Knockdown of AhR results in cardiac hypertrophy and specific AhR-knock-down in vascular endothelial cells cause hypotension [85, 86]. Furthermore, overexpression of AhR has been shown to induce endothelial dysfunction [87]. AhR expression and polymorphisms were also associated with risk of coronary arterial disease in a Chinese population [88]. Compared with controls, blood levels of AhR were found to be significantly increased in patients with coronary arterial disease [88]. In line with this, DEP-exposure has been reported to induce cardiac dysfunction and remodeling (left ventricular dilation) through an AhR-dependent mechanism [89]. Furthermore, the prototypical environmental AhR ligand, 3,4,7,8-tetrachlorodibenzo-*p*-dioxin (TCDD) has been reported to induce cardiomyopathies, cardiac lesions, arteritis, and atherosclerosis in rodents, and increase the risk of CVD in humans [83]. Recently it was also shown that TCDD inhibits cardiomyocyte differentiation from human embryonic stem cells via AhR-regulated mechanisms [90].

### Calcium signaling

The cytosolic concentration of calcium [Ca^2+^]_*i*_ is central to pathophysiological processes including AhR-genomic signaling, oxidative stress and inflammation [91, 92]. In endothelial cells [Ca^2+^]_*i*_ regulates blood pressure and flow, especially through control of vascular smooth muscle cells via myo-endothelial micro-domains and eNOS [93–96]. Furthermore, [Ca^2+^]_*i*_ is involved in regulation of endothelial permeability, a central step in the pathogenesis of atherosclerosis [97, 98].

Activation of Ca^2+^-channels in the plasma membrane such as transient receptor potential (TRP) channels, results in Ca^2+^-influx [99]. Notably, a number of studies suggest that combustion particles including DEP and wood smoke particles, and chemicals attached may trigger health effects by affecting Ca^2+^ flux through TRP-channels [100, 101]. Some of the TRP-channels appear to be activated through direct interaction with particles or attached chemicals, while others seem to be activated by more indirect mechanisms such as transactivation. Importantly, several TRP-channels are central to endothelial homeostasis, and seem to play a role in development of CVD, especially by affecting endothelial function [102–104].

[Ca^2+^]_*i*_ is also regulated via Ca^2+^-release from intracellular stores such as the endoplasmic reticulum or mitochondria. This may result from activating G protein-coupled receptors (GPCRs) or receptor tyrosine kinases (RTKs) [105, 106]. β1- and β2-adrenergic receptors (ADRs) regulate cardiopulmonary function and immune responses, and are among the main drug-targets in CVD treatment [107–109]. Certain PAHs known to be present in DEP may increase [Ca^2+^]_*i*_ in human micro-vascular endothelial cells (HMEC-1) and other cell types via β2ADRs [110–112]. In human bronchial epithelial BEAS-2B cells exposed to 1-nitropyrene (1-NP), β2ADRs appeared to be involved in [Ca^2+^]_*i*_-increase and induction of the pro-inflammatory cytokine CXCL8 [111].

Transporters, channels and receptors cluster in membrane micro domains [113], and their activity may also be affected by their presence inside or outside such ordered domains [114, 115]. Several xenobiotics including DEP-extracts and PAHs have been found to affect membrane microstructure, thus possibly affecting [Ca^2+^]_*i*_ or other signaling mechanisms by altering the membrane physiology [116–119].

## Search strategy and review structure

As a starting point the following search terms were used in PubMed: (((“Cardiovascular Diseases”[Mesh]) OR “Blood Pressure”[Mesh])) AND ((((((“Air Pollutants”[Mesh]) OR “Air Pollution”[Mesh]) OR “Environmental Exposure”[Mesh]) OR “Inhalation Exposure/adverse effects”[Mesh])) AND “Polycyclic Aromatic Hydrocarbons”[Mesh]) (29.5.2018). Using this approach 121 studies were found. Only 12 of these studies were linked to general population when excluding studies on health effects of cancer therapy (eg. with anthracyclines) and occupation. Thus, we additionally included occupational studies of environmental setting to the papers reviewed. Studies of PAH at high non-environmental settings (e.g. coke oven workers) were also commented as they were regarded to present relevant information.

Given the difficulty of identifying relevant animal and in vitro mechanistic studies linking PAH to CVD from other literature, additional strategies were also used. A number of searches were performed in PubMed using combinations PAH or specific PAH and terms linked to CVD including endothelial dysfunction, foam cells and cardiovascular development. Some papers were identified by tracking the citation network (cited and citing papers) of identified papers, while some were from the authors personal databases.

Publications identified were screened at abstract level. A total of 19 epidemiological studies exploring cardiovascular effects of exposure to environmental levels of PAHs and CVD were included. No formal analysis of these studies was however undertaken.

With regard to available animal and mechanistic research, we highlight research suggesting that extractable organic material of combustion particles, PAHs and AhR and intracellular calcium could be linked to cellular processes central in development and exacerbation of CVD. Concentrations or exposure routes used in experimental studies with pure PAH-exposure were not evaluated. Information from these studies were included to explore possible mechanisms involved and added as proof of principle.

## The role of organic chemicals and PAH in mediating CVD

### Human exposure and epidemiological studies

Exposure to PM_2.5_/DEP has been found to cause dysfunction of cells and biological processes of the cardiovascular system linked to CVD, including atherosclerosis, hypertension, myocardial infarction, stroke, thrombosis and restricted valve motion (Table 3) [3, 4]. Furthermore, accumulating evidence suggests that PM/DEP with the highest portion of organic chemicals have the greatest effects on vascular outcomes [2, 11, 35, 120, 121]. A recent review reported that most epidemiological studies found significant positive association between PAHs exposure and manifest CVD, as well as major risk factors predisposing for CVD including elevated blood pressure [122].

**Table 3:**
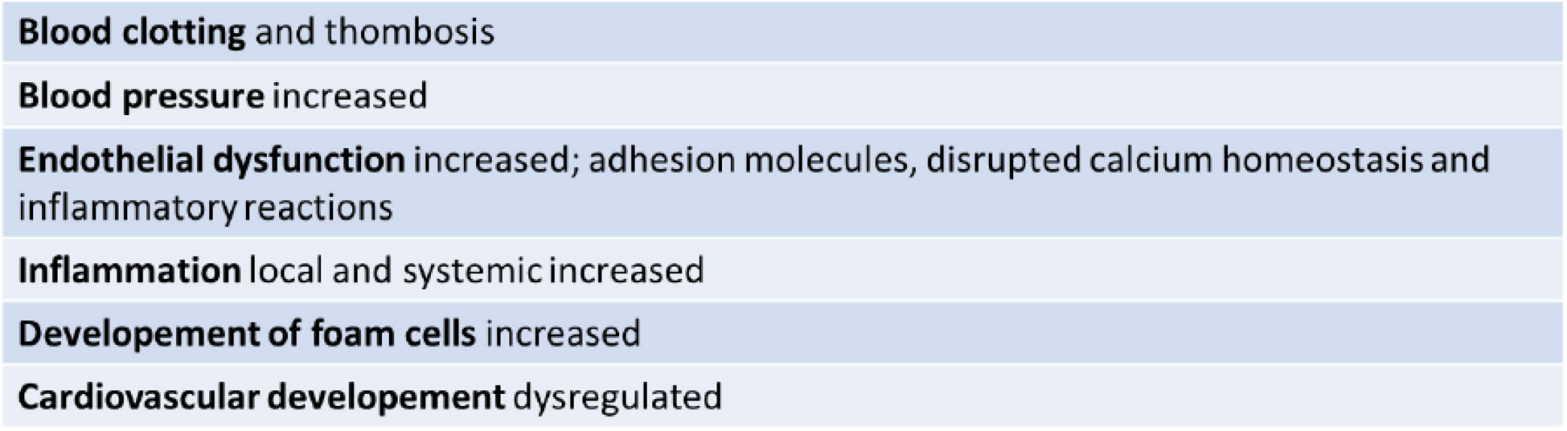
Effects of combustion particles/PAHs on processes linked to CVD

Importantly, we are not only exposed to PAHs through polluted air. As reviewed elsewhere tobacco smoke and foods are among the major sources in addition to occupational exposures [21]. The relative contribution of PAHs from air pollution versus other sources with regard to CVD will depend on the location, activity and dietary habits of the population in study. However, the majority of PAHs absorbed via the gastro-intestinal tract will go through first-path metabolism and elimination in the liver. By contrast, it has been shown by Gerde et al. [40] that inhaled B[*a*]P taken up through the alveolar region primarily enters the circulation, reaching the heart and vasculature in an un-metabolized state. Thus, the importance of air pollution as a source for circulatory levels of parent PAHs should not be underestimated.

Urinary 1-hydroxypyrene, a metabolite of pyrene, is among the most commonly used biomarkers. Although 1-hydroxypyrene concentrations are correlated to smoking, certain PAH-rich food items and occupational exposure studies have shown that there is a statistically significant correlation between urinary 1-hydroxypyrene concentrations and ambient air levels of pyrene and benzo[*a*]pyrene (B[*a*]P), in subjects that smoke less than 20 cigarettes daily [21]. Thus, it has been argued that 1-hydroxypyrene is a valid biomarker also of PAH exposure from ambient air.

#### Heart disease and mortality rates

PM and PAH exposures may occur in occupational settings at levels 1–3 orders of magnitude higher than those in environmental settings [123]. Notably, heart disease mortality rates in occupational cohorts such as aluminum smelters are typically lower than those in the general population [124, 125], likely due to the “healthy worker effect” bias which has been suggested to be strong for diseases of the cardiovascular system [126]. The relation between exposure to PAH and mortality from ischemic heart disease (IHD; 418 cases) was studied in a cohort of 12,367 male asphalt workers from various nations. Both cumulative and average exposure indices for B[*a*]P were positively associated with mortality, and demonstrated a consistent exposure–response relation for this association [127]. Recent morbidity studies among aluminum smelters have reported associations of adverse cardiovascular effects with PM and PAH exposure, by using biomarkers of CVD, such as markers of inflammation, blood pressure, and heart rate variability. Ischemic heart disease mortality was associated with B[*a*]P in the highest exposure category. A monotonic, but non-significant trend was observed between chronic B[*a*]P exposure and acute myocardial infarction. When follow-up was restricted to active employment, hazard ratio for ischemic heart disease was 2.39 in the highest cumulative B[*a*]P category. The stronger associations observed during employment suggests that risk may not persist after exposure cessation [128].

In a cohort of autoworkers, modest evidence that occupational exposure to PM_3.5_ containing PAHs may increase risk of ischemic heart disease mortality was reported [129]. In a population-based case-reference study of myocardial infarction and occupational exposure to motor exhaust and other combustion products, relative risk of myocardial infarction was 2.11 among highly exposed and 1.42 among those intermediately exposed to combustion products from organic material. Furthermore, exposure-response patterns in terms of both maximum exposure intensity and cumulative dose, were found [130].

Exposure to traffic increased the risk of myocardial infarction in susceptible subjects [131]. Increased onset of chest pain was observed immediately and 6 h after traffic exposure. Exacerbated heart symptoms among myocardial infarction survivors have been linked to particle-associated organic compounds [132]. The study showed an association between PM, adhered organic chemicals and daily symptoms.

The association between particle-associated PAH concentrations and symptom-severity among myocardial infarction survivors, suggests a major influence on cardiovascular health [133]. In the general population, cross-sectional studies have reported that concentration of urinary mono-hydroxy metabolites of phenanthrene, was significantly associated with self-reported CVD [19]. Subjects with middle and highest tertile of fluorene and phenanthrene metabolites had a similar significantly higher prevalence of peripheral arterial disease as compared to subjects within the lowest tertile [134]. A Chinese multi-provincial cohort study found an association between the 10-year risk of atherosclerotic cardiovascular disease and PAHs exposure measured as urinary OH-PAH metabolites, more specifically: 2-hydroxyfluoren, 9-hydroxyfluoren, 1-hydroxyphenanthrene and ΣOH-PAHs levels [135]. Increased serum C-reactive protein (CRP) has also been reported from subjects with elevated levels of urinary PAHs, indicating inflammation [136].

Occupational PAH exposure among boilermakers [137, 138] and coke oven workers [137] has been associated with altered heart rate variability. Recently, an association between background PAH exposure and heart rate variability in the general population was also reported. Increased urinary OH-PAH metabolites and Framingham risk scores were dose-dependently related to decreased heart rate variability [139].

#### Inflammatory markers

Urinary 1-hydroxypyrene was increased among taxi-drivers compared to non-occupationally exposed subjects, and positively correlated with pro-inflammatory cytokines (IL-1β, IL-6, IL-10, TNF-α, IFN-γ and hs-CRP) and biomarkers of oxidative damage (serum levels of oxidized LDL, auto-antibodies and homocysteine), but negatively correlated with antioxidants. As higher levels of inflammatory biomarkers and homocysteine, represent important predictors for cardiovascular events, these data suggest a possible link between PAH exposures and CVD in an occupational setting [140]. In support of this, a study comparing taxi drivers with and without co-morbidity found that urinary 1-hydroxypyrene levels were associated with carotid intima-media thickness and with serum homocysteine levels [141]. Occupational exposure to soot, rich in PAH, has also been associated with increased risk of CVD. In a cross-sectional study of chimney sweepers, it was found that chimney sweepers had up to 7-fold higher concentrations of PAH metabolites in urine than controls. Furthermore, early markers of CVD, namely homocysteine, cholesterol, and reduced levels of high density lipoproteins (HDL), were affected in serum from chimney sweepers. PAH metabolites correlated positively with the percentage of time spent soot sweeping. A study of school-age children identified increases in oxidative stress biomarkers in association with urinary PAHs [138].

#### Blood pressure

Coke oven workers are highly exposed to PAHs, and studies have associated coke oven emission to hypertension and abnormal electrocardiography [142–144]. Furthermore, the levels of 2-hydroxyphenanthrene, 3-hydroxybenzo[*a*]pyrene, and 3-hydroxy-benzo[*a*]anthracene were positively associated with diastolic blood pressure in chimney sweepers [145]. Contrasting findings were reported in a study of blood pressure changes and exposure to PAHs in outdoor workers. The results showed that in the total group, levels of urinary 1-hydroxypyrene were negatively associated with systolic and diastolic blood pressure [146]. The authors suggest that occupational exposure to PAHs may significantly influence blood pressure, probably acting via the autonomic nervous system [146].

In a population of elderly, the oxy-PAHs, chrysene-5,6-dione and B[*a*]P-3,6-dione were significantly associated with increased systolic blood pressure and pulse pressure [147]. PAHs were also associated with hypertension in a study among relatively young individuals, mostly women [148]. An association between PAHs in housewives’ hair and hypertension has been investigated [149]. Seven PAHs in hair samples were measured with detection rate > 70%. Only acenaphthylene was found to be associated with an increased risk of hypertension. Among children in Saudi Arabia, proximity to an oil refinery was associated with prehypertension and increased PAH and PM exposure. However, urinary PAH metabolites (1-hydroxypyrene and hydroxyphenanthrene) were not associated with cardiovascular outcomes in the study [150]. In a repeated measures study of 106 residents in China, eight urinary PAHs metabolites (OH-PAHs) were analyzed. Association of OH-PAHs with high blood pressure and increased risk for atherosclerotic CVD were found, and was suggested to be partially mediated by obesity [151].

#### Embryonic development

Passive diffusion seems to control trans-placental transfer of PAHs from maternal serum to the fetal circulation [152]. There is evidence from experimental systems that exposure to PAHs results in congenital heart defects [153], as discussed later. However, results from a large population-based study do not support an association between potential maternal occupational exposure to PAHs and various congenital heart defects [154].

#### Evaluation of epidemiological studies

Overall, the epidemiological data suggest a possible association between PAH-exposure and CVD, although some inconsistencies have been reported amongst others for hypertension. Furthermore, the number of available studies is limited, and the role of PAHs can be difficult to disentangle from other combustion-related pollutants, especially in non-occupational cohort studies. It should also be emphasized that no formal analysis of the strengths of these epidemiological studies was performed in this review. Thus, conclusions should not be drawn based on these epidemiological data alone.

### Experimental studies in animals and mechanistic considerations

In vivo animal studies addressing cardiovascular effects of PM were recently summarized by Møller and coworkers [155]. Some challenges regarding extrapolation from animal studies to humans were discussed. Atherosclerosis is not as common in rodents as in man, e.g. wildtype mice do not develop atherosclerosis; even in the often used atherosclerosis-prone ApoE^−/−^ (knockdown) mouse model, plaques formed do not rupture. In humans plaque rupture is a pivotal event in acute myocardial infarction and stroke [155].

#### Effects of DEP on CVD considering content of organic chemicals

As animal models seem to be less sensitive, quantitative consideration based on these studies are less relevant. However, they do give important knowledge with regard to possible mechanisms involved. Most studies on the widely used automobile-derived DEP sample A-DEP (50% organic chemicals) reported effects on either atheroma development or vasomotor function, as reviewed by Møller et al. [155]. ApoE^−/−^ mice exposed to SRM 1650 (20% organic chemicals), had increased plaques progression [156]. Studies with SRM 2975, which has a low content of organic chemicals (5%) [157, 158], are somewhat contradictory and less convincing. In Wistar rats exposed to SRM 2975 by intra-tracheal instillation, vasomotor function was unaffected although the rats had extensive lung inflammation [159]. However, SRM 2975 disturbed vasomotor function of hypertensive rats [160]. Furthermore, SRM 2975 has been reported to increase both lung inflammation as well as plaque formation in ApoE^−/−^ mice [60].

Several studies on DEP with various content of organic chemicals have suggested that they are important triggers of effects on CVD. Compared to PM_2.5_, ultrafine particles contained more than twice the amount of organic chemicals, and induced significantly more pro-atherogenic effects in vivo [35]. Keebaugh and co-workers have possibly conducted the most compelling in vivo evidence that organic chemicals attached to combustion particles is important for atherosclerosis development [43]. ApoE^−/−^ mice exposed to concentrated ambient particles (CAPs) had accelerated atherosclerosis development and reduced cardiac function, while these responses in mice exposed to CAPs denuded of organic chemicals were not statistically different from mice exposed to air.

#### Effects on heart

PAHs such as B[*a*]P are known to bind to and activate the AhR, leading to increased expression of xenobiotic metabolic enzymes (XME) such as CYP1A1, CYP1A2, CYP1B1, NAD(P)H:quinone oxidoreductase-1 (NQO1), and glutathione S-transferase A1 (GSTA1) [68], enhanced production of ROS and reactive PAH species leading to lipid peroxidation and tissue damage. Notably, studies from our lab and others, suggest that AhR-activation and induction of CYP1-expression may be the most sensitive endpoint in DEP- and PM-exposed cells [161, 162]. AhR and AhR-regulated xenobiotic metabolizing enzymes appear to be highly expressed in the cardiovascular system. The various AhR-regulated genes are differentially expressed in different parts. Particularly high levels are found in the endothelium of aorta, coronary arteries and ventricles [163]. Disruption of synthesis and/or metabolism of endogenous substances, such as arachidonic acid (AA), prostaglandins (PGs), and thyroid hormones has been suggested to contribute to the pathogenesis of CVD [164]. Studies in rats and chicken embryos have demonstrated that CYP1A1 mediates metabolism of arachidonic acid to hydroxyeicosatetraenoic acid (HETE), epoxyeicosatrienoic acid (EET), and Prostaglandin E2 metabolites [165, 166]. These endogenous substances may thus be targets for PAH-mediated cardiotoxicity via AhR-induced metabolism. While cardiac AhR-regulated CYPs are involved in CVD pathogenesis; the AhR-regulated enzymes NQO1 and GST are regarded as more cardio-protective. An imbalance in expression of cardio-toxic and cardio-protective xenobiotic metabolizing enzymes has thus been suggested as a main determinant of PAH-mediated cardiotoxicity [163]. However, as AhR also appear to play a central endogenous role in development and homeostasis of the cardiovascular system, impact of PAHs on CVD is likely not restricted to regulation of xenobiotic metabolism.

Experimental studies support the epidemiological findings indicating that exposure to AhR agonists in the environment may lead to cardiovascular toxicity. AhR agonists such as 3-methylcholantrene (3-MC) and B[*a*]P increased the heart to body weight ratio as well as levels of hypertrophic markers, such as atrial natriuretic peptide and brain natriuretic peptide in rats [167]. In contrast, treatment with B[*e*]P, an isomer of B[*a*]P that is a poor AhR-ligand, did not induce cardiac hypertrophy, supporting a role of AhR in the pathogenesis of cardiac hypertrophy. However, phenanthrene, which is also generally considered a poor activator of AhR-mediated CYP-1 expression, was reported to induce cardiac hypertrophy through a mechanism involving reduced miR-133 expression [168]. In the same study, effects on cardiomyocyte size and protein content in vitro, was observed at low nanomolar concentrations of phenanthrene (0.05–0.5 nM). Additionally, other AhR-ligands [163, 169] have been reported to cause cardio-toxic effects in humans and experimental animals. Exposure to B[*a*]P, induced atherosclerosis to a greater extent in mice with high-affinity AhR (B6) and had a huge impact on gene expression of the aorta when compared to mice with low affinity AhR (B6.D2) [170]. This study also indicated an endogenous role of AhR signaling in regulating heart size.

Genome-wide association studies have identified susceptibility loci and candidate genetic variants that predispose to coronary artery disease in humans [171]. Such studies have identified variation at 6q23.2 to be associated with this disease in Caucasian and Han Chinese populations [172], and identified *TCF21* as the causal gene in this locus [173]. Recent findings suggest that TCF21 and AhR cooperate to activate a pro-inflammatory gene expression program in coronary artery smooth muscle cells to activate an inflammatory gene expression program that is exacerbated by TCDD or PAHs, and may contribute to the overall risk for coronary arterial disease [71]. Furthermore, the study showed that AhR gene expression was increased in atherosclerotic lesions in mice, using laser capture microdissection. The AhR protein was localized in human carotid atherosclerotic lesions, where it was associated with protein kinases with a critical role in innate immune responses.

#### Oxidative stress, inflammation and atherosclerosis

There are studies suggesting that PAHs like B[*a*]P aggravate atherosclerosis via increased oxidative stress [174]. Oxidative stress in the vascular endothelium will decrease availability of NO, a key regulator of vascular tone and blood pressure. Overexpression of antioxidant enzymes in ApoE-deficient mice suppressed B[*a*]P-accelerated atherosclerosis [6]. Some studies find that B[*a*]P and other AhR agonists increase expression of pro-inflammatory cytokines such as CXCL8 and TNF-α, which in turn cause infiltration of macrophages and neutrophils into the lungs [73]. Pulmonary oxidative stress and inflammation may result in “systemic spill-over” suggested to be important for development of CVD [3].

Oxidative stress may also lead to alteration in circulating lipids. Elevations of serum cholesterol-associated triglyceride and LDL are regarded as primary causative factors for the development of atherosclerosis. These pro-atherogenic molecules diffuse into sub-endothelial cells and cause further vascular dysfunction [2]. Microarray studies after TCDD exposure in rodents revealed AhR involvement in regulation of fatty acid and cholesterol synthesis [175]. In humans, industrial exposure to dioxin has been associated with lipid metabolism disruption and high levels of circulating cholesterol and triglyceride [176].

Atherosclerosis induced by PAHs has similarly been linked to AhR-dependent effects on cholesterol synthesis [177]. Furthermore, B[*a*]P-exposed ApoE^−/−^ mice show enhanced monocyte chemoattractant protein-1 (MCP-1) gene expression and marked increase of macrophage content in atherosclerotic plaques [178]. Furthermore, in vitro studies have found that B[a]P-induced MCP-1 gene and protein expression was inhibited by AhR antagonism, but not antioxidant treatment, suggesting AhR-mediated induction [179]. B[a]P also enhanced TNFα-induced expression of MCP-1, thus supporting the concept that induction of inflammation is a crucial process in PAH-enhanced atherogenesis.

#### DNA damage and atherosclerosis

Environmental chemical carcinogens including PAHs have also been suggested as risk factor for atherosclerosis as a result of their mutagenic effects [180, 181]. Atherosclerosis is associated with DNA damage in both circulating and vessel-wall cells. Furthermore, DNA adducts derived from exposure to environmental mutagens are abundant in atherosclerotic vessels, and PAH-DNA damage has been detected in human endothelial and smooth vascular muscle cells [182]. It has been suggested that PAHs could cause aberrant smooth muscle cell proliferation, a focal point in the genesis and progression of atherosclerosis [183–185]. As seen in smooth muscle cells isolated from B[*a*]P-treated quail, serial sub-culture of smooth muscle cells exposed to B[*a*]P in vitro yielded a fast-growing population of cells after the fifth passage [183]. The pathogenic relevance of mutation-related molecular damage in atherosclerosis has only partial support in experimental animal models. Both the number and size of arterial lesions in the brachiocephalic arteries in atherosclerosis-susceptible (“White Carneau”) and atherosclerosis-resistant (“Show Racer”) females pigeons were significantly enhanced after long-term dosing with B[*a*]P [186]. Exposure to B[*e*]P did not enhance lesion development, and no effects were observed in males. On the other hand, chronic exposure to B[*a*]P only induced larger and phenotypically different atherosclerotic plaques in ApoE-knockout mice while their location or number seemed unaffected [187]. Furthermore, morphometrical analysis show that B[*e*]P causes a similar increase in plaque size. Thus, in some models it seems that PAHs may induce an inflammatory atherosclerotic plaque phenotype irrespective of DNA- and/or AhR-binding properties [178].

#### Endothelial dysfunction

Endothelial dysfunction is a key initiating event in numerous CVD including atherosclerosis. Endothelial cells have the highest induction of CYP1A1 in the vasculature of rat models, and these cells have ability to metabolize B[*a*]P [188]. The vascular endothelium is susceptible to damage, as it is in constant contact with circulating xenobiotics including PAHs. Accordingly, B[*a*]P-DNA adducts have been found in the endothelium from human atherosclerotic lesions [182].

In vitro studies indicate that DEP may impair endothelial function [62]. Dysfunction of the endothelium is marked by increased adhesiveness caused by the presentation of cellular adhesion molecules, such as intercellular adhesion molecule 1 (ICAM-1) and vascular cell adhesion molecule-1 (VCAM-1). These are essential initiating events of atherosclerosis, which result in retention of macrophages and monocytes in the sub-endothelial space. DEP have been shown to increase endothelial cell permeability through down-regulation of tight junction proteins, decreased trans-endothelial resistance and redistribution of vascular endothelial cadherin from cell membrane intracellularly [189]. Data from in vitro studies on human endothelial cells demonstrate that B[*a*]P is able to increase ICAM-1 through a caveolae- and AhR-mediated pathway, thereby increasing monocyte adhesion [190].

Endothelial dysfunction is also characterized by disruption of calcium homeostasis [93, 95] and increased expression of pro-inflammatory markers [191]. Interestingly, in a human exposure study, the calcium blocker verapamil lost its vasodilatory effect after exposure to DEP, indicating effects on [Ca^2+^]_*i*_ regulation [192]. In a recent in vitro study, DEP applied on the epithelial side of an alveolar 3D tri-culture, rapidly induced pro-inflammatory and AhR-regulated genes in basolateral endothelial cells [48]. Further analysis of endothelial cells showed that lipophilic extracts of DEP containing most of the PAHs, modified calcium homeostasis and increased expression of pro-inflammatory markers via an AhR-dependent pathway [48, 116]. The authors hypothesized that PAHs detached from DEP translocate through alveolar epithelial cells and modify calcium homeostasis and increase inflammatory reactions, thereby triggering endothelial dysfunction.

In human endothelial HMEC-1 cells, B[a]P triggered a relatively rapid and transient increase of [Ca^2+^]_*i*_, which was prevented by co-treatment with β2ADR inhibitors, anti-β2ADR antibodies, or siRNA-mediated knockdown of β2ADR expression [112]. B[*a*]P was shown, to bind β2ADR directly with high affinity (Kd = 10 nM), as assessed by in vitro binding assays and molecular modeling [112]. This interaction also appear to cause a desensitization of β2ADR signaling, leading to reduced responsiveness towards epinephrine [193]. In vitro studies on lung epithelial cells suggest that 1-nitropyrene may induce Ca^2+^-signaling at least partly through activation of β2ADR, and that both β2ADR- and Ca^2+^-signaling may be involved in CXCL8 up-regulation [111]. However, the 1-nitropyrene concentration required to activate β2ADR-induced Ca^2+^-signaling, appeared to be an order of magnitude higher than B[*a*]P [111]. Other G protein-coupled receptors (GPCRs) have also been implicated in DEP-induced Ca^2+^-signaling and inflammation. Li et al. [100] found that organic chemicals extracted from DEP increased [Ca^2+^]_*i*_ via the GPCR, protease activated receptor 2 (PAR-2), in primary human bronchial cells. PARs are central for regulating vascular function, and activation may promote conversion of endothelial cells into a pro-inflammatory phenotype in conditions associated with endothelial dysfunction [194]. Recently it was reported that both induction of inflammation-associated genes and [Ca^2+^]_*i*_, partly depended on PAR-2 in endothelial cells exposed to lipophilic organic chemicals from DEP [48, 195].

Pyrene and B[*e*]P, two PAHs normally considered to have low affinity for the AhR, also induced marked [Ca^2+^]_*i*_ induction in the HMEC-1 endothelial cell line. Especially pyrene appeared to be a potent inducer of Ca^2+^-signaling, triggering approximately 2-fold higher [Ca^2+^]_*i*_-responses compared to B[*a*]P or chrysene [112]. Surprisingly, pyrene-induced [Ca^2+^]_i_ seemed to depend on a selective activation of non-genomic AhR-signaling, occurring in absence of effects on CYP1A1 expression [119]. This differs distinctly from B[*a*]P-induced Ca^2+^-signaling through β2ADR [112], and suggest that PAHs may affect [Ca^2+^]_*i*_-regulation in endothelial cell through different mechanisms. Notably, studies on effect of lipophilic organic chemicals extracted from DEP, suggest that their induction of [Ca^2+^]_*i*_ could involve both AhR- and β2ADR-dependent responses [119, 195].

Phenanthraquinone, another PAH-derivative found in DEP, has a potent inhibitory effect on eNOS activity [196]. TCDD was also reported to suppress eNOS, but induced iNOS [197]. Moreover, increased eNOS activity and enhanced vessel elasticity was observed in AhR-deficient mice, while AhR overexpression ex vivo suppressed the migratory capacity of endothelial cells, impaired eNOS activation and suppressed NO production [198]. Furthermore, AhR was reported to be involved in cigarette smoke-induced increases in carotid intimal thickening in mice, through activation of iNOS expression in smooth muscle cells [199]. Notably, eNOS levels are reduced and iNOS levels increased during endothelial aging and dysfunction [200]. Thus, it has been speculated that increased AhR expression could result in vascular inflammation through induction of iNOS and thereby further contribute to endothelial dysfunction and vessel aging [198].

#### Effects on blood pressure

Exposure to PAHs in experimental animals has produced equivocal effects on hypertension. 3-MC-induced hypertension was found to be associated with eNOS inactivation [201]. NO produced by endothelial cells is a key regulator of vascular tone and blood pressure. Thus, an inactivation of eNOS may cause suppression of NO-mediated vaso-relaxation and elevation of blood pressure. In another study, intranasal B[*a*]P was found to alter circadian blood pressure patterns and cause lung inflammation [202]. Atherosclerosis and hypertension are risk factors for development of abdominal aortic aneurysms. Thus, it is interesting to note that B[*a*]P aggravates development of abdominal aortic aneurysms in ApoE-knockout mice [203]. Furthermore, ischemia-induced angiogenesis by B[*a*]P was found to depend on AhR [204].

#### Development of foam cells

Air pollution particles increased formation of lipid loaded foam cells from macrophages in vitro [64]. The formation of foam cells in sub-endothelial lesions play a key role in atherogenesis, in part by releasing pro-inflammatory cytokines. Most interestingly, in vitro studies with the human macrophage cell line U937 showed that organic extracts from ultrafine particles (UFP) and DEP as well as TCDD induced more expression of CXCL8, TNF-α, and COX-2 mRNA, than stripped particles, whereas total particles (parent and stripped particles) led to a greater production of C-reactive protein and IL-6 mRNA [64]. UFP-, DEP- and organic extract-induced expressions of COX-2 and CYP1A1 were suppressed by AhR antagonist, indicating that these effects are mainly mediated by organic components which can activate the AhR. In contrast, induction of C-reactive protein and IL-6 seem to be particle-related effects that are AhR-independent. The inflammatory responses induced by PM and their respective organic extracts were also associated with a subsequent increase of cholesterol accumulation. B[*a*]P has been found to cause similar lipid accumulation in primary human macrophages obtained by GM-CSF-mediated differentiation of monocytes [205]. The effect was linked to an AhR-dependent repression of Niemann–Pick type C1 protein (NPC1). Interestingly, NPC1 is a transmembrane protein containing a sterol-sensitive domain that participates in cholesterol trafficking from the late endosome/lysosome to the plasma membrane, and is therefore thought to protect cells from intracellular accumulation of cholesterol [206]. Taken together, these data illustrate that vascular inflammatory responses as well as foam cell-formation caused by PM, are tightly linked to organic chemicals triggering AhR responses. Even though foam cell formation caused by PM is likely triggered by several components, PAHs seem to represent possible candidates.

#### Embryonic development

Several studies indicate that in utero exposure to PAHs dysregulate cardiovascular development. Following in utero B[*a*]P exposure of Long Evans Hooded rats, systolic blood pressure was significantly elevated in offspring on postnatal day P53 in the middle and high exposure groups as compared to controls [207]. A number of studies have also investigated effects of PM and PAHs on cardiovascular development in zebrafish embryos. PM-induced disruption of cardiovascular development in zebrafish embryos has been suggested in part to be due to AhR-mediated suppression of Wnt-signaling, and has been attributed to PAHs [208–210]. Defects in cardiac function preceded characteristic morphological abnormalities induced in zebrafish embryos exposed to a mixture of PAHs [153]. Most interestingly, the relative toxicity of different mixtures was directly proportional to the amount of phenanthrene, or dibenzothiophene-phenanthrene total in the mixture, apparently due to direct effects on cardiac conduction.

Pyrene induced a different syndrome of anemia, peripheral vascular defects, and neuronal cell death in zebrafish, similar to effects previously described for potent AhR ligands [153]. Embryonic exposure of zebrafish to pyrene, has also been shown to disrupt normal cardiac development resulting in heart abnormalities, such as pericardial edema and cardiac looping defects, and alter expression of defective cardiac differentiation-related genes [211]. Furthermore, in zebrafish embryos exposed to phenanthrene it has also been observed abnormally looped and enlarged hearts along with increased expression of matrix metalloproteinase-9 (MMP-9) and transforming growth factor [212]. Low level co-exposure with silica nanoparticles and B[*a*]P induced inflammatory response and hyper-coagulable conditions in zebrafish embryos [213]. Thus it appears that different PAH species may have distinct and specific effects on early cardiovascular development in fish. It seems that these effects are due to mechanisms that differ from classical AhR-ligand binding and/or formation of electrophiles.

Although existence of high-affinity physiological activators of AhR are still debated, also endogenous signaling of the AhR is believed to be important in the development and function of cardiovascular system, based on studies with *AhR* gene-deficient mice [83]. These studies show that AhR-knockout mice develop cardiac hypertrophy, abnormal vascular structure in multiple organs and altered blood pressure depending on their host environment and point to a role for genetic variations of this gene.

## Conclusion

Human epidemiological studies combined with experimental studies strongly suggest that exposure to combustion particles enhance risk of CVD, including atherosclerosis, hypertension, thrombosis and myocardial infarction. Overall, epidemiological studies both in occupational settings and the general population suggest possible associations between environmental PAH exposure and CVD including well known CVD-risk factors. However, it should be noted that the literature is limited and some inconsistencies have been reported.

Animal models seem less sensitive than epidemiological studies, but combined with in vitro experimental models they increase our understanding of possible biological mechanisms through which exposure to PM may cause adverse effects linked to CVD. Experiments suggest that organic compounds attached to combustion particles are of importance for triggering CVD, and furthermore that their effects are mediated at least in part by AhR. This is in accordance with a role of PAHs, a well-known group of chemicals present on combustion particles which bind to AhR and/or are metabolically activated by CYP-enzymes.

A number of cardiovascular effects of TCDD and AhR knock-down or overexpression are now well documented, highlighting the central role of AhR in CVD. Specific studies with PAHs show that also B[*a*]P is cardio-toxic, and increased the heart to body weight ratio as well as levels of hypertrophy markers via AhR-dependent mechanisms. Likewise, certain PAHs may produce equivocal effects on hypertension in experimental animals. Furthermore, PAHs accelerate development of atherosclerosis, induce major changes in gene expression depending on AhR, and B[*a*]P DNA adducts are found in atherosclerotic lesions.

There are studies that support mechanisms of cardiovascular toxicity involving AhR, ROS and/or reactive electrophilic metabolites. On the other hand, it seems as PAHs in some models may induce an inflammatory atherosclerotic plaque phenotype irrespective of their DNA- and/or AhR-ligand binding properties. Importantly, cardiovascular effects of PAHs may not be restricted to B[*a*]P, but has also been reported for pyrene, phenanthrene and B[*e*]P. Additionally, animal knock-out studies clearly link AhR as such to CVD, pointing to the possibility that exposure to PAH may disturb AhR-regulated gene-expression linked to endogenous ligands important for a well-functioning cardiovascular system.

There is still a need to expand our knowledge on the role of PM-composition for development and/or exacerbation of CVD. The key drivers of cardiovascular effects observed in combustion-PM exposed populations still remains to be clearly identified. Nevertheless, mechanistic studies in animals and cell models suggest that PAHs adhered to combustion particles may be among the critical determinants in CVD. This notion seem to be supported by epidemiological studies. Several uncertainties regarding the suggested mechanisms involved and importance of different PAH species remain to be elucidated, and studies assessing the association between PAHs and CVD in the general population remains scarce. This warrants further studies as enhanced knowledge may have implication for risk assessment of combustion particles and their associated PAHs.

## Data Availability

“Not applicable”.

## Abbreviations

[Ca^2+^]_*i*_: Cytosolic concentration of calcium
ADRs: Adrenergic receptors
AhR: Aryl hydrocarbon receptor
ARNT: AhR nuclear translocator
B[*a*]P: Benzo[*a*]pyrene
CVD: Cardiovascular diseases
CYP: Cytochrome P450
DEP: Diesel exhaust particles
HDL: High density lipoproteins
NF-κB: Nuclear factor-κB
oxLDL: Oxidized low density lipoproteins
PAHs: Polycyclic aromatic hydrocarbons
PM: Particular matter
ROS: Reactive oxygen species
TRP: Transient receptor potential
WHO: World Health Organization

## References

[1] WHO (2016). Ambient air pollution: a global assesment of exposure and burden of disease.

[2] Lee KK, Miller MR, Shah ASV (2018). Air pollution and stroke. J Stroke.

[3] Brook RD, Rajagopalan S, Pope CA, Brook JR, Bhatnagar A, Diez-Roux AV, Holguin F, Hong Y, Luepker RV, Mittleman MA (2010). Particulate matter air pollution and cardiovascular disease: an update to the scientific statement from the American Heart Association. Circulation.

[4] Lelieveld Jos, Klingmüller Klaus, Pozzer Andrea, Pöschl Ulrich, Fnais Mohammed, Daiber Andreas, Münzel Thomas (2019). Cardiovascular disease burden from ambient air pollution in Europe reassessed using novel hazard ratio functions. European Heart Journal.

[5] Pope CA, Turner MC, Burnett RT, Jerrett M, Gapstur SM, Diver WR, Krewski D, Brook RD (2015). Relationships between fine particulate air pollution, cardiometabolic disorders, and cardiovascular mortality. Circ Res.

[6] Cohen AJ, Brauer M, Burnett R, Anderson HR, Frostad J, Estep K, Balakrishnan K, Brunekreef B, Dandona L, Dandona R (2017). Estimates and 25-year trends of the global burden of disease attributable to ambient air pollution: an analysis of data from the global burden of diseases study 2015. Lancet.

[7] Cassee FR, Heroux ME, Gerlofs-Nijland ME, Kelly FJ (2013). Particulate matter beyond mass: recent health evidence on the role of fractions, chemical constituents and sources of emission. Inhal Toxicol.

[8] Lewtas J (2007). Air pollution combustion emissions: characterization of causative agents and mechanisms associated with cancer, reproductive, and cardiovascular effects. Mutat Res.

[9] Snider G, Weagle CL, Murdymootoo KK, Ring A, Ritchie Y, Stone E, Walsh A, Akoshile C, Anh NX, Balasubramanian R (2016). Variation in global chemical composition of emerging results from SPARTAN. Atmos Chem Phys.

[10] Grahame TJ, Klemm R, Schlesinger RB (2014). Public health and components of particulate matter: the changing assessment of black carbon. J Air Waste Manage Assoc.

[11] Araujo JA, Nel AE (2009). Particulate matter and atherosclerosis: role of particle size, composition and oxidative stress. Part Fibre Toxicol.

[12] Kittelson DB (1998). Engines and nanoparticles: a review. J Aerosol Sci.

[13] Matti Maricq M (2007). Chemical characterization of particulate emissions from diesel engines: a review. J Aerosol Sci.

[14] Scheer V, Kirchner U, Casati R, Vogt R, Philippin S, Wiedensohler A, Hock N, Schneider J, Weimer S, Borrmann S (2005). Composition of semi-volatile particles from diesel exhaust.

[15] NTP DoHaHSa. Diesel exhaust particulates. In*.* Edited by Services DoHaH. 2011. Available from: http://ntp.niehs.nih.gov/ntp/roc/content/profiles/dieselexhaustparticulates.pdf.

[16] Wichmann HE (2007). Diesel exhaust particles. Inhal Toxicol.

[17] Bostrom CE, Gerde P, Hanberg A, Jernstrom B, Johansson C, Kyrklund T, Rannug A, Tornqvist M, Victorin K, Westerholm R (2002). Cancer risk assessment, indicators, and guidelines for polycyclic aromatic hydrocarbons in the ambient air. Environ Health Perspect.

[18] Javed W, Iakovides M, Stephanou EG, Wolfson JM, Koutrakis P, Guo B (2019). Concentrations of aliphatic and polycyclic aromatic hydrocarbons in ambient PM2.5 and PM10 particulates in Doha, Qatar. J Air Waste Manage Assoc (1995).

[19] Xu X, Cook RL, Ilacqua VA, Kan H, Talbott EO, Kearney G (2010). Studying associations between urinary metabolites of polycyclic aromatic hydrocarbons (PAHs) and cardiovascular diseases in the United States. Sci Total Environ.

[20] Shen H, Huang Y, Wang R, Zhu D, Li W, Shen G, Wang B, Zhang Y, Chen Y, Lu Y (2013). Global atmospheric emissions of polycyclic aromatic hydrocarbons from 1960 to 2008 and future predictions. Environ Sci Technol.

[21] Srogi K (2007). Monitoring of environmental exposure to polycyclic aromatic hydrocarbons: a review. Environ Chem Lett.

[22] Gualtieri M, Ovrevik J, Holme JA, Perrone MG, Bolzacchini E, Schwarze PE, Camatini M (2010). Differences in cytotoxicity versus pro-inflammatory potency of different PM fractions in human epithelial lung cells. Toxicol in Vitro.

[23] Siponen T, Yli-Tuomi T, Aurela M, Dufva H, Hillamo R, Hirvonen MR, Huttunen K, Pekkanen J, Pennanen A, Salonen I (2015). Source-specific fine particulate air pollution and systemic inflammation in ischaemic heart disease patients. Occup Environ Med.

[24] Schneider A, Neas LM, Graff DW, Herbst MC, Cascio WE, Schmitt MT, Buse JB, Peters A, Devlin RB (2010). Association of cardiac and vascular changes with ambient PM2.5 in diabetic individuals. Part Fibre Toxicol.

[25] Pope CA, Bhatnagar A, McCracken JP, Abplanalp W, Conklin DJ, O'Toole T (2016). Exposure to fine particulate air pollution is associated with endothelial injury and systemic inflammation. Circ Res.

[26] Giorgini P, Di Giosia P, Grassi D, Rubenfire M, Brook RD, Ferri C (2016). Air pollution exposure and blood pressure: an updated review of the literature. Curr Pharm Des.

[27] Krishnan RM, Adar SD, Szpiro AA, Jorgensen NW, Van Hee VC, Barr RG, O'Neill MS, Herrington DM, Polak JF, Kaufman JD (2012). Vascular responses to long- and short-term exposure to fine particulate matter: MESA air (multi-ethnic study of atherosclerosis and air pollution). J Am Coll Cardiol.

[28] Cosselman KE, Krishnan RM, Oron AP, Jansen K, Peretz A, Sullivan JH, Larson TV, Kaufman JD (2012). Blood pressure response to controlled diesel exhaust exposure in human subjects. Hypertension.

[29] Van Eeden S, Leipsic J, Paul Man SF, Sin DD (2012). The relationship between lung inflammation and cardiovascular disease. Am J Respir Crit Care Med.

[30] Mills NL, Tornqvist H, Gonzalez MC, Vink E, Robinson SD, Soderberg S, Boon NA, Donaldson K, Sandstrom T, Blomberg A (2007). Ischemic and thrombotic effects of dilute diesel-exhaust inhalation in men with coronary heart disease. N Engl J Med.

[31] Mills NL, Tornqvist H, Robinson SD, Gonzalez M, Darnley K, MacNee W, Boon NA, Donaldson K, Blomberg A, Sandstrom T (2005). Diesel exhaust inhalation causes vascular dysfunction and impaired endogenous fibrinolysis. Circulation.

[32] Lucking AJ, Lundback M, Mills NL, Faratian D, Barath SL, Pourazar J, Cassee FR, Donaldson K, Boon NA, Badimon JJ (2008). Diesel exhaust inhalation increases thrombus formation in man. Eur Heart J.

[33] Carll AP, Hazari MS, Perez CM, Krantz QT, King CJ, Winsett DW, Costa DL, Farraj AK (2012). Whole and particle-free diesel exhausts differentially affect cardiac electrophysiology, blood pressure, and autonomic balance in heart failure-prone rats. Toxicol Sci.

[34] Kodavanti UP (2016). Stretching the stress boundary: linking air pollution health effects to a neurohormonal stress response. Biochim Biophys Acta.

[35] Araujo JA, Barajas B, Kleinman M, Wang X, Bennett BJ, Gong KW, Navab M, Harkema J, Sioutas C, Lusis AJ (2008). Ambient particulate pollutants in the ultrafine range promote early atherosclerosis and systemic oxidative stress. Circ Res.

[36] Borm PJ, Robbins D, Haubold S, Kuhlbusch T, Fissan H, Donaldson K, Schins R, Stone V, Kreyling W, Lademann J (2006). The potential risks of nanomaterials: a review carried out for ECETOC. Part Fibre Toxicol.

[37] Miller MR, Raftis JB, Langrish JP, McLean SG, Samutrtai P, Connell SP, Wilson S, Vesey AT, Fokkens PHB, Boere AJF (2017). Inhaled nanoparticles accumulate at sites of vascular disease. ACS Nano.

[38] Miller MR, Raftis JB, Langrish JP, McLean SG, Samutrtai P, Connell SP, Wilson S, Vesey AT, Fokkens PHB, Boere AJF (2017). Correction to “inhaled nanoparticles accumulate at sites of vascular disease”. ACS Nano.

[39] Penn A, Murphy G, Barker S, Henk W, Penn L (2005). Combustion-derived ultrafine particles transport organic toxicants to target respiratory cells. Environ Health Perspect.

[40] Gerde P, Muggenburg BA, Lundborg M, Dahl AR (2001). The rapid alveolar absorption of diesel soot-adsorbed benzo[a]pyrene: bioavailability, metabolism and dosimetry of an inhaled particle-borne carcinogen. Carcinogenesis.

[41] Ovrevik J, Refsnes M, Lag M, Brinchmann BC, Schwarze PE, Holme JA (2017). Triggering mechanisms and inflammatory effects of combustion exhaust particles with implication for carcinogenesis. Basic Clin Pharmacol Toxicol.

[42] Gutleb AC (2011). Potential of in vitro methods for mechanistic studies of particulate matter–induced cardiopulmonary toxicity. Crit Rev Environ Sci Technol.

[43] Keebaugh AJ, Sioutas C, Pakbin P, Schauer JJ, Mendez LB, Kleinman MT (2015). Is atherosclerotic disease associated with organic components of ambient fine particles?. Sci Total Environ.

[44] Kawasaki S, Takizawa H, Takami K, Desaki M, Okazaki H, Kasama T, Kobayashi K, Yamamoto K, Nakahara K, Tanaka M (2001). Benzene-extracted components are important for the major activity of diesel exhaust particles. Am J Respir Cell Mol Biol.

[45] Bonvallot V, Baeza-Squiban A, Baulig A, Brulant S, Boland S, Muzeau F, Barouki R, Marano F (2001). Organic compounds from diesel exhaust particles elicit a proinflammatory response in human airway epithelial cells and induce cytochrome p450 1A1 expression. Am J Respir Cell Mol Biol.

[46] Totlandsdal AI, Herseth JI, Bolling AK, Kubatova A, Braun A, Cochran RE, Refsnes M, Ovrevik J, Lag M (2012). Differential effects of the particle core and organic extract of diesel exhaust particles. Toxicol Lett.

[47] Ma JY, Ma JK (2002). The dual effect of the particulate and organic components of diesel exhaust particles on the alteration of pulmonary immune/inflammatory responses and metabolic enzymes. J Environ Sci Health C Environ Carcinog Ecotoxicol Rev.

[48] Brinchmann BC, Skuland T, Rambol MH, Szoke K, Brinchmann JE, Gutleb AC, Moschini E, Kubatova A, Kukowski K, Le Ferrec E (2018). Lipophilic components of diesel exhaust particles induce pro-inflammatory responses in human endothelial cells through AhR dependent pathway(s). Part Fibre Toxicol.

[49] van der Vorst EP, Doring Y, Weber C (2015). Chemokines and their receptors in atherosclerosis. J Mol Med (Berl).

[50] McLaren JE, Michael DR, Ashlin TG, Ramji DP (2011). Cytokines, macrophage lipid metabolism and foam cells: implications for cardiovascular disease therapy. Prog Lipid Res.

[51] Ramji DP, Davies TS (2015). Cytokines in atherosclerosis: key players in all stages of disease and promising therapeutic targets. Cytokine Growth Factor Rev.

[52] Donaldson K, Stone V, Seaton A, MacNee W (2001). Ambient particle inhalation and the cardiovascular system: potential mechanisms. Environ Health Perspect.

[53] Maier KL, Alessandrini F, Beck-Speier I, Hofer TP, Diabate S, Bitterle E, Stoger T, Jakob T, Behrendt H, Horsch M (2008). Health effects of ambient particulate matter--biological mechanisms and inflammatory responses to in vitro and in vivo particle exposures. Inhal Toxicol.

[54] de Kok TM, Driece HA, Hogervorst JG, Briede JJ (2006). Toxicological assessment of ambient and traffic-related particulate matter: a review of recent studies. Mutat Res.

[55] Donaldson K, Stone V, Borm PJA, Jimenez LA, Gilmour PS, Schins RPF, Knaapen AM, Rahman I, Faux SP, Brown DM (2003). Oxidative stress and calcium signaling in the adverse effects of environmental particles (PM10). Free Radic Biol Med.

[56] Li N, Hao M, Phalen RF, Hinds WC, Nel AE (2003). Particulate air pollutants and asthma. A paradigm for the role of oxidative stress in PM-induced adverse health effects. Clin Immunol (Orlando, Fla).

[57] Sauer H, Wartenberg M, Hescheler J (2001). Reactive oxygen species as intracellular messengers during cell growth and differentiation. Cell Physiol Biochem.

[58] Ovrevik J, Refsnes M, Lag M, Holme JA, Schwarze PE (2015). Activation of proinflammatory responses in cells of the airway mucosa by particulate matter: oxidant- and non-oxidant-mediated triggering mechanisms. Biomolecules.

[59] Tornqvist H, Mills NL, Gonzalez M, Miller MR, Robinson SD, Megson IL, Macnee W, Donaldson K, Soderberg S, Newby DE (2007). Persistent endothelial dysfunction in humans after diesel exhaust inhalation. Am J Respir Crit Care Med.

[60] Miller MR, McLean SG, Duffin R, Lawal AO, Araujo JA, Shaw CA, Mills NL, Donaldson K, Newby DE, Hadoke PW (2013). Diesel exhaust particulate increases the size and complexity of lesions in atherosclerotic mice. Part Fibre Toxicol.

[61] Forchhammer L, Loft S, Roursgaard M, Cao Y, Riddervold IS, Sigsgaard T, Moller P (2012). Expression of adhesion molecules, monocyte interactions and oxidative stress in human endothelial cells exposed to wood smoke and diesel exhaust particulate matter. Toxicol Lett.

[62] Lawal AO, Zhang M, Dittmar M, Lulla A, Araujo JA (2015). Heme oxygenase-1 protects endothelial cells from the toxicity of air pollutant chemicals. Toxicol Appl Pharmacol.

[63] Tseng CY, Wang JS, Chao MW (2017). Causation by diesel exhaust particles of endothelial dysfunctions in cytotoxicity, pro-inflammation, permeability, and apoptosis induced by ROS generation. Cardiovasc Toxicol.

[64] Vogel CFA, Sciullo E, Wong P, Kuzmicky P, Kado N, Matsumura F (2005). Induction of proinflammatory cytokines and C-reactive protein in human macrophage cell line U937 exposed to air pollution particulates. Environ Health Perspect.

[65] Barouki R, Aggerbeck M, Aggerbeck L, Coumoul X (2012). The aryl hydrocarbon receptor system. Drug Metabol Drug Interact.

[66] Esser C, Rannug A (2015). The aryl hydrocarbon receptor in barrier organ physiology, immunology, and toxicology. Pharmacol Rev.

[67] Namazi MR (2009). Cytochrome-P450 enzymes and autoimmunity: expansion of the relationship and introduction of free radicals as the link. J Autoimmune Dis.

[68] Miller KP, Ramos KS (2001). Impact of cellular metabolism on the biological effects of benzo[a]pyrene and related hydrocarbons. Drug Metab Rev.

[69] Tian Y (2002). Ah receptor and NF-kB interactions: mechanisms and physiological implications. Chem Biol Interact.

[70] Fardel O (2013). Cytokines as molecular targets for aryl hydrocarbon receptor ligands: implications for toxicity and xenobiotic detoxification. Expert Opin Drug Metab Toxicol.

[71] Kim JB, Pjanic M, Nguyen T, Miller CL, Iyer D, Liu B, Wang T, Sazonova O, Carcamo-Orive I, Matic LP (2017). TCF21 and the environmental sensor aryl-hydrocarbon receptor cooperate to activate a pro-inflammatory gene expression program in coronary artery smooth muscle cells. PLoS Genet.

[72] N'Diaye M, Le Ferrec E, Lagadic-Gossmann D, Corre S, Gilot D, Lecureur V, Monteiro P, Rauch C, Galibert MD, Fardel O (2006). Aryl hydrocarbon receptor- and calcium-dependent induction of the chemokine CCL1 by the environmental contaminant benzo[a]pyrene. J Biol Chem.

[73] Podechard N, Lecureur V, Le Ferrec E, Guenon I, Sparfel L, Gilot D, Gordon JR, Lagente V, Fardel O (2008). Interleukin-8 induction by the environmental contaminant benzo(a)pyrene is aryl hydrocarbon receptor-dependent and leads to lung inflammation. Toxicol Lett.

[74] Tian Y, Rabson AB, Gallo MA (2002). Ah receptor and NF-kappaB interactions: mechanisms and physiological implications. Chem Biol Interact.

[75] Vogel CF, Matsumura F (2009). A new cross-talk between the aryl hydrocarbon receptor and RelB, a member of the NF-kappaB family. Biochem Pharmacol.

[76] Denison MS, Soshilov AA, He G, DeGroot DE, Zhao B (2011). Exactly the same but different: promiscuity and diversity in the molecular mechanisms of action of the aryl hydrocarbon (dioxin) receptor. Toxicol Sci.

[77] Matsumura F (2009). The significance of the nongenomic pathway in mediating inflammatory signaling of the dioxin-activated Ah receptor to cause toxic effects. Biochem Pharmacol.

[78] Tomkiewicz C, Herry L, Bui LC, Metayer C, Bourdeloux M, Barouki R, Coumoul X (2013). The aryl hydrocarbon receptor regulates focal adhesion sites through a non-genomic FAK/Src pathway. Oncogene.

[79] Lawal AO (2017). Air particulate matter induced oxidative stress and inflammation in cardiovascular disease and atherosclerosis: the role of Nrf2 and AhR-mediated pathways. Toxicol Lett.

[80] Yi T, Wang J, Zhu K, Tang Y, Huang S, Shui X, Ding Y, Chen C, Lei W (2018). Aryl hydrocarbon receptor: a new Player of pathogenesis and therapy in cardiovascular diseases. Biomed Res Int.

[81] Xiao L, Zhang Z, Luo X (2014). Roles of xenobiotic receptors in vascular pathophysiology. Circ J.

[82] Korashy HM, El-Kadi AOS (2008). The role of aryl hydrocarbon receptor in the pathogenesis of cardiovascular diseases. Drug Metab Rev.

[83] Zhang N (2011). The role of endogenous aryl hydrocarbon receptor signaling in cardiovascular physiology. J Cardiovasc Dis Res.

[84] Mohsenzadeh MS, Zanjani BR, Karimi G (2018). Mechanisms of 2,3,7,8-tetrachlorodibenzo-p-dioxin- induced cardiovascular toxicity: an overview. Chem Biol Interact.

[85] Agbor LN, Elased KM, Walker MK (2011). Endothelial cell-specific aryl hydrocarbon receptor knockout mice exhibit hypotension mediated, in part, by an attenuated angiotensin II responsiveness. Biochem Pharmacol.

[86] Vasquez A, Atallah-Yunes N, Smith FC, You X, Chase SE, Silverstone AE, Vikstrom KL (2003). A role for the aryl hydrocarbon receptor in cardiac physiology and function as demonstrated by AhR knockout mice. Cardiovasc Toxicol.

[87] Eckers A, Sauerbier E, Anwar-Mohamed A, Hamann I, Esser C, Schroeder P, El-Kadi AO, Klotz LO (2011). Detection of a functional xenobiotic response element in a widely employed FoxO-responsive reporter construct. Arch Biochem Biophys.

[88] Huang S, Shui X, He Y, Xue Y, Li J, Li G, Lei W, Chen C (2015). AhR expression and polymorphisms are associated with risk of coronary arterial disease in Chinese population. Sci Rep.

[89] Bradley JM, Cryar KA, El Hajj MC, El Hajj EC, Gardner JD (2013). Exposure to diesel exhaust particulates induces cardiac dysfunction and remodeling. J Appl Physiol (Bethesda, Md: 1985).

[90] Fu H, Wang L, Wang J, Bennett BD, Li JL, Zhao B, Hu G (2019). Dioxin and AHR impairs mesoderm gene expression and cardiac differentiation in human embryonic stem cells. Sci Total Environ.

[91] Racioppi L, Means AR (2012). Calcium/calmodulin-dependent protein kinase kinase 2: roles in signaling and pathophysiology. J Biol Chem.

[92] Gorlach A, Bertram K, Hudecova S, Krizanova O (2015). Calcium and ROS: a mutual interplay. Redox Biol.

[93] Sandow SL, Senadheera S, Grayson TH, Welsh DG, Murphy TV (2012). Calcium and endothelium-mediated vasodilator signaling. Adv Exp Med Biol.

[94] Moller P, Mikkelsen L, Vesterdal LK, Folkmann JK, Forchhammer L, Roursgaard M, Danielsen PH, Loft S (2011). Hazard identification of particulate matter on vasomotor dysfunction and progression of atherosclerosis. Crit Rev Toxicol.

[95] Sandow SL, Haddock RE, Hill CE, Chadha PS, Kerr PM, Welsh DG, Plane F (2009). What’s where and why at a vascular myoendothelial microdomain signalling complex. Clin Exp Pharmacol Physiol.

[96] Clapham DE (2007). Calcium signaling. Cell.

[97] Tiruppathi C, Minshall RD, Paria BC, Vogel SM, Malik AB (2002). Role of Ca2+ signaling in the regulation of endothelial permeability. Vasc Pharmacol.

[98] Tiruppathi C, Ahmmed GU, Vogel SM, Malik AB (2006). Ca2+ signaling, TRP channels, and endothelial permeability. Microcirculation.

[99] Ramsey IS, Delling M, Clapham DE (2006). An introduction to TRP channels. Annu Rev Physiol.

[100] Li J, Kanju P, Patterson M, Chew WL, Cho SH, Gilmour I, Oliver T, Yasuda R, Ghio A, Simon SA (2011). TRPV4-mediated calcium influx into human bronchial epithelia upon exposure to diesel exhaust particles. Environ Health Perspect.

[101] Fariss MW, Gilmour MI, Reilly CA, Liedtke W, Ghio AJ (2013). Emerging mechanistic targets in lung injury induced by combustion-generated particles. Toxicol Sci.

[102] Dietrich A, Kalwa H, Gudermann T (2010). TRPC channels in vascular cell function. Thromb Haemost.

[103] Smedlund K, Bah M, Vazquez G (2012). On the role of endothelial TRPC3 channels in endothelial dysfunction and cardiovascular disease. Cardiovasc Hematol Agents Med Chem.

[104] Earley S, Brayden JE (2015). Transient receptor potential channels in the vasculature. Physiol Rev.

[105] Altier C (2012). GPCR and voltage-gated calcium channels (VGCC) signaling complexes. Subcell Biochem.

[106] Zamponi GW (2015). Calcium channel signaling complexes with receptors and channels. Curr Mol Pharmacol.

[107] De Backer G (2003). European guidelines on cardiovascular disease prevention in clinical practice third joint task force of European and other societies on cardiovascular disease prevention in clinical practice (constituted by representatives of eight societies and by invited experts). Eur Heart J.

[108] Kolmus K, Tavernier J, Gerlo S (2015). beta2-Adrenergic receptors in immunity and inflammation: stressing NF-kappaB. Brain Behav Immun.

[109] Wachter SB, Gilbert EM (2012). Beta-adrenergic receptors, from their discovery and characterization through their manipulation to beneficial clinical application. Cardiology.

[110] Mayati A, Le Ferrec E, Lagadic-Gossmann D, Fardel O (2012). Aryl hydrocarbon receptor-independent up-regulation of intracellular calcium concentration by environmental polycyclic aromatic hydrocarbons in human endothelial HMEC-1 cells. Environ Toxicol.

[111] Mayati A, Le Ferrec E, Holme JA, Fardel O, Lagadic-Gossmann D, Ovrevik J (2014). Calcium signaling and beta2-adrenergic receptors regulate 1-nitropyrene induced CXCL8 responses in BEAS-2B cells. Toxicol in Vitro.

[112] Mayati A, Levoin N, Paris H, N'Diaye M, Courtois A, Uriac P, Lagadic-Gossmann D, Fardel O, Le Ferrec E (2012). Induction of intracellular calcium concentration by environmental benzo(a)pyrene involves a beta2-adrenergic receptor/adenylyl cyclase/Epac-1/inositol 1,4,5-trisphosphate pathway in endothelial cells. J Biol Chem.

[113] Bastiani M, Parton RG (2010). Caveolae at a glance. J Cell Sci.

[114] Isshiki M, Anderson RG (2003). Function of caveolae in Ca2+ entry and Ca2+−dependent signal transduction. Traffic (Copenhagen, Denmark).

[115] Santos AL, Preta G (2018). Lipids in the cell: organisation regulates function. Cell Mol Life Sci.

[116] Brinchmann Bendik, Le Ferrec Eric, Podechard Normand, Lagadic-Gossmann Dominique, Shoji Kenji, Penna Aubin, Kukowski Klara, Kubátová Alena, Holme Jørn, Øvrevik Johan (2018). Lipophilic Chemicals from Diesel Exhaust Particles Trigger Calcium Response in Human Endothelial Cells via Aryl Hydrocarbon Receptor Non-Genomic Signalling. International Journal of Molecular Sciences.

[117] Tekpli X, Holme JA, Sergent O, Lagadic-Gossmann D (2013). Role for membrane remodeling in cell death: implication for health and disease. Toxicology.

[118] Podechard N, Chevanne M, Fernier M, Tete A, Collin A, Cassio D, Kah O, Lagadic-Gossmann D, Sergent O (2017). Zebrafish larva as a reliable model for in vivo assessment of membrane remodeling involvement in the hepatotoxicity of chemical agents. J Appl Toxicol.

[119] Brinchmann BC, Ferrec EL, Bisson WH, Podechard N, Huitfeldt HS, Gallais I, Sergent O, Holme JA, Lagadic-Gossmann D, Øvrevik J (2018). Evidence of selective activation of aryl hydrocarbon receptor nongenomic calcium signaling by pyrene. Biochem Pharmacol.

[120] Ntziachristos L, Froines JR, Cho AK, Sioutas C (2007). Relationship between redox activity and chemical speciation of size-fractionated particulate matter. Part Fibre Toxicol.

[121] Pope CA, Dockery DW (2006). Health effects of fine particulate air pollution: lines that connect. J Air Waste Manage Assoc (1995).

[122] Poursafa P, Moosazadeh M, Abedini E, Hajizadeh Y, Mansourian M, Pourzamani H, Amin MM (2017). A systematic review on the effects of polycyclic aromatic hydrocarbons on cardiometabolic impairment. Int J Prev Med.

[123] Liu B, Jia C (2016). Effects of profession on urinary PAH metabolite levels in the US population. Int Arch Occup Environ Health.

[124] Ronneberg A (1995). Mortality and cancer morbidity in workers from an aluminium smelter with prebaked carbon anodes--part III: mortality from circulatory and respiratory diseases. Occup Environ Med.

[125] Moulin JJ, Clavel T, Buclez B, Laffitte-Rigaud G (2000). A mortality study among workers in a French aluminium reduction plant. Int Arch Occup Environ Health.

[126] Thygesen LC, Hvidtfeldt UA, Mikkelsen S, Bronnum-Hansen H (2011). Quantification of the healthy worker effect: a nationwide cohort study among electricians in Denmark. BMC Public Health.

[127] Burstyn I, Kromhout H, Partanen T, Svane O, Langard S, Ahrens W, Kauppinen T, Stucker I, Shaham J, Heederik D (2005). Polycyclic aromatic hydrocarbons and fatal ischemic heart disease. Epidemiology.

[128] Friesen MC, Demers PA, Spinelli JJ, Eisen EA, Lorenzi MF, Le ND (2010). Chronic and acute effects of coal tar pitch exposure and cardiopulmonary mortality among aluminum smelter workers. Am J Epidemiol.

[129] Costello S, Garcia E, Hammond SK, Eisen EA (2013). Ischemic heart disease mortality and PM(3.5) in a cohort of autoworkers. Am J Ind Med.

[130] Gustavsson P, Plato N, Hallqvist J, Hogstedt C, Lewne M, Reuterwall C, Scheele P (2001). A population-based case-referent study of myocardial infarction and occupational exposure to motor exhaust, other combustion products, organic solvents, lead, and dynamite. Stockholm heart epidemiology program (SHEEP) study group. Epidemiology.

[131] Peters A, von Klot S, Heier M, Trentinaglia I, Hormann A, Wichmann HE, Lowel H (2004). Exposure to traffic and the onset of myocardial infarction. N Engl J Med.

[132] Kraus U, Breitner S, Schnelle-Kreis J, Cyrys J, Lanki T, Ruckerl R, Schneider A, Bruske I, Gu J, Devlin R (2011). Particle-associated organic compounds and symptoms in myocardial infarction survivors. Inhal Toxicol.

[133] Schnelle-Kreis J, Kupper U, Sklorz M, Cyrys J, Briede JJ, Peters A, Zimmermann R (2009). Daily measurement of organic compounds in ambient particulate matter in Augsburg, Germany: new aspects on aerosol sources and aerosol related health effects. Biomarkers.

[134] Xu X, Hu H, Kearney GD, Kan H, Sheps DS (2013). Studying the effects of polycyclic aromatic hydrocarbons on peripheral arterial disease in the United States. Sci Total Environ.

[135] Hu C, Hou J, Zhou Y, Sun H, Yin W, Zhang Y, Wang X, Wang G, Chen W, Yuan J (2018). Association of polycyclic aromatic hydrocarbons exposure with atherosclerotic cardiovascular disease risk: a role of mean platelet volume or club cell secretory protein. Environ Pollut.

[136] Everett CJ, King DE, Player MS, Matheson EM, Post RE, Mainous AG (2010). Association of urinary polycyclic aromatic hydrocarbons and serum C-reactive protein. Environ Res.

[137] Li X, Feng Y, Deng H, Zhang W, Kuang D, Deng Q, Dai X, Lin D, Huang S, Xin L (2012). The dose-response decrease in heart rate variability: any association with the metabolites of polycyclic aromatic hydrocarbons in coke oven workers?. PLoS One.

[138] Lee MS, Magari S, Christiani DC (2011). Cardiac autonomic dysfunction from occupational exposure to polycyclic aromatic hydrocarbons. Occup Environ Med.

[139] Feng Y, Sun H, Song Y, Bao J, Huang X, Ye J, Yuan J, Chen W, Christiani DC, Wu T (2014). A community study of the effect of polycyclic aromatic hydrocarbon metabolites on heart rate variability based on the Framingham risk score. Occup Environ Med.

[140] Brucker N, Moro AM, Charao MF, Durgante J, Freitas F, Baierle M, Nascimento S, Gauer B, Bulcao RP, Bubols GB (2013). Biomarkers of occupational exposure to air pollution, inflammation and oxidative damage in taxi drivers. Sci Total Environ.

[141] Brucker N, Charao MF, Moro AM, Ferrari P, Bubols G, Sauer E, Fracasso R, Durgante J, Thiesen FV, Duarte MM (2014). Atherosclerotic process in taxi drivers occupationally exposed to air pollution and co-morbidities. Environ Res.

[142] Liang JJ, Yi GL, Mao GS, Wang DM, Dai XY (2016). Influence of coke oven emissions on workers’ blood pressure and electrocardiographic findings. Zhonghua Lao Dong Wei Sheng Zhi Ye Bing Za Zhi.

[143] Sroczynski J, Biskupek K, Podolecki A, Schneiberg P (1990). Effect of work in the coke-producing plant on the circulatory system of workers. Med Pr.

[144] Yang K, Jiang X, Cheng S, Chen C, Cao X, Tu B (2017). Effects of coke oven emissions and benzo[a]pyrene on blood pressure and electrocardiogram in coke oven workers. J Occup Health.

[145] Alhamdow A, Lindh C, Albin M, Gustavsson P, Tinnerberg H, Broberg K (2017). Early markers of cardiovascular disease are associated with occupational exposure to polycyclic aromatic hydrocarbons. Sci Rep.

[146] Sancini A, Caciari T, Sinibaldi F, Sacco C, Boscolo P, Giubilati R, Scala B, Tomei G, Tomei F, Rosati MV (2014). Blood pressure changes and polycyclic aromatic hydrocarbons in outdoor workers. Clin Ter.

[147] Jacobs L, Buczynska A, Walgraeve C, Delcloo A, Potgieter-Vermaak S, Van Grieken R, Demeestere K, Dewulf J, Van Langenhove H, De Backer H (2012). Acute changes in pulse pressure in relation to constituents of particulate air pollution in elderly persons. Environ Res.

[148] Bangia KS, Symanski E, Strom SS, Bondy M (2015). A cross-sectional analysis of polycyclic aromatic hydrocarbons and diesel particulate matter exposures and hypertension among individuals of Mexican origin. Environ Health.

[149] Wang B, Li Z, Ma Y, Qiu X, Ren A (2016). Association of polycyclic aromatic hydrocarbons in housewives’ hair with hypertension. Chemosphere.

[150] Trasande L, Urbina EM, Khoder M, Alghamdi M, Shabaj I, Alam MS, Harrison RM, Shamy M (2015). Polycyclic aromatic hydrocarbons, brachial artery distensibility and blood pressure among children residing near an oil refinery. Environ Res.

[151] Yin W, Hou J, Xu T, Cheng J, Li P, Wang L, Zhang Y, Wang X, Hu C, Huang C (2018). Obesity mediated the association of exposure to polycyclic aromatic hydrocarbon with risk of cardiovascular events. Sci Total Environ.

[152] Zhang X, Li X, Jing Y, Fang X, Zhang X, Lei B, Yu Y (2017). Transplacental transfer of polycyclic aromatic hydrocarbons in paired samples of maternal serum, umbilical cord serum, and placenta in Shanghai, China. Environ Pollut.

[153] Incardona JP, Collier TK, Scholz NL (2004). Defects in cardiac function precede morphological abnormalities in fish embryos exposed to polycyclic aromatic hydrocarbons. Toxicol Appl Pharmacol.

[154] Lupo PJ, Symanski E, Langlois PH, Lawson CC, Malik S, Gilboa SM, Lee LJ, Agopian AJ, Desrosiers TA, Waters MA (2012). Maternal occupational exposure to polycyclic aromatic hydrocarbons and congenital heart defects among offspring in the national birth defects prevention study. Birth Defects Res A Clin Mol Teratol.

[155] Moller P, Christophersen DV, Jacobsen NR, Skovmand A, Gouveia AC, Andersen MH, Kermanizadeh A, Jensen DM, Danielsen PH, Roursgaard M (2016). Atherosclerosis and vasomotor dysfunction in arteries of animals after exposure to combustion-derived particulate matter or nanomaterials. Crit Rev Toxicol.

[156] Poss J, Lorenz D, Werner C, Pavlikova V, Gensch C, Speer T, Alessandrini F, Berezowski V, Kuntz M, Mempel M (2013). Diesel exhaust particles impair endothelial progenitor cells, compromise endothelial integrity, reduce neoangiogenesis, and increase atherogenesis in mice. Cardiovasc Toxicol.

[157] DeMarini DM, Brooks LR, Warren SH, Kobayashi T, Gilmour MI, Singh P (2004). Bioassay-directed fractionation and salmonella mutagenicity of automobile and forklift diesel exhaust particles. Environ Health Perspect.

[158] Singh P, DeMarini DM, Dick CA, Tabor DG, Ryan JV, Linak WP, Kobayashi T, Gilmour MI (2004). Sample characterization of automobile and forklift diesel exhaust particles and comparative pulmonary toxicity in mice. Environ Health Perspect.

[159] Robertson S, Gray GA, Duffin R, McLean SG, Shaw CA, Hadoke PW, Newby DE, Miller MR (2012). Diesel exhaust particulate induces pulmonary and systemic inflammation in rats without impairing endothelial function ex vivo or in vivo. Part Fibre Toxicol.

[160] Labranche N, El Khattabi C, Dewachter L, Dreyfuss C, Fontaine J, van de Borne P, Berkenboom G, Pochet S (2012). Vascular oxidative stress induced by diesel exhaust microparticles: synergism with hypertension. J Cardiovasc Pharmacol.

[161] Totlandsdal AI, Cassee FR, Schwarze P, Refsnes M, Lag M (2010). Diesel exhaust particles induce CYP1A1 and pro-inflammatory responses via differential pathways in human bronchial epithelial cells. Part Fibre Toxicol.

[162] Andrysik Z, Vondracek J, Marvanova S, Ciganek M, Neca J, Pencikova K, Mahadevan B, Topinka J, Baird WM, Kozubik A (2011). Activation of the aryl hydrocarbon receptor is the major toxic mode of action of an organic extract of a reference urban dust particulate matter mixture: the role of polycyclic aromatic hydrocarbons. Mutat Res.

[163] Korashy HM, El-Kadi AO (2006). The role of aryl hydrocarbon receptor in the pathogenesis of cardiovascular diseases. Drug Metab Rev.

[164] Rifkind AB, Gannon M, Gross SS (1990). Arachidonic acid metabolism by dioxin-induced cytochrome P-450: a new hypothesis on the role of P-450 in dioxin toxicity. Biochem Biophys Res Commun.

[165] Annas A, Brunstrom B, Brandt I, Brittebo EB (1998). Induction of ethoxyresorufin O-deethylase (EROD) and endothelial activation of the heterocyclic amine Trp-P-1 in bird embryo hearts. Arch Toxicol.

[166] Quilley CP, Rifkind AB (1986). Prostaglandin release by the chick embryo heart is increased by 2,3,7,8-tetrachlorodibenzo-p-dioxin and by other cytochrome P-448 inducers. Biochem Biophys Res Commun.

[167] Aboutabl ME, Zordoky BN, El-Kadi AO (2009). 3-methylcholanthrene and benzo(a)pyrene modulate cardiac cytochrome P450 gene expression and arachidonic acid metabolism in male Sprague Dawley rats. Br J Pharmacol.

[168] Huang L, Xi Z, Wang C, Zhang Y, Yang Z, Zhang S, Chen Y, Zuo Z (2016). Phenanthrene exposure induces cardiac hypertrophy via reducing miR-133a expression by DNA methylation. Sci Rep.

[169] Kopf PG, Huwe JK, Walker MK (2008). Hypertension, cardiac hypertrophy, and impaired vascular relaxation induced by 2,3,7,8-tetrachlorodibenzo-p-dioxin are associated with increased superoxide. Cardiovasc Toxicol.

[170] Kerley-Hamilton JS, Trask HW, Ridley CJA, DuFour E, Lesseur C, Ringelberg CS, Moodie KL, Shipman SL, Korc M, Gui J (2012). Inherent and benzo[a]pyrene-induced differential aryl hydrocarbon receptor signaling greatly affects life span, atherosclerosis, cardiac gene expression, and body and heart growth in mice. Toxicol Sci.

[171] Schunkert H, König IR, Kathiresan S, Reilly MP, Assimes TL, Holm H, Preuss M, Stewart AFR, Barbalic M, Gieger C (2011). Large-scale association analysis identifies 13 new susceptibility loci for coronary artery disease. Nat Genet.

[172] Wang Y, Wang L, Liu X, Zhang Y, Yu L, Zhang F, Liu L, Cai J, Yang X, Wang X (2014). Genetic variants associated with myocardial infarction and the risk factors in Chinese population. PLoS One.

[173] Miller CL, Anderson DR, Kundu RK, Raiesdana A, Nürnberg ST, Diaz R, Cheng K, Leeper NJ, Chen C-H, Chang IS (2013). Disease-related growth factor and embryonic signaling pathways modulate an enhancer of TCF21 expression at the 6q23.2 coronary heart disease locus. PLoS Genetics.

[174] Yang H, Zhou L, Wang Z, Roberts LJ, Lin X, Zhao Y, Guo Z (2009). Overexpression of antioxidant enzymes in ApoE-deficient mice suppresses benzo(a)pyrene-accelerated atherosclerosis. Atherosclerosis.

[175] Sato S, Shirakawa H, Tomita S, Ohsaki Y, Haketa K, Tooi O, Santo N, Tohkin M, Furukawa Y, Gonzalez FJ (2008). Low-dose dioxins alter gene expression related to cholesterol biosynthesis, lipogenesis, and glucose metabolism through the aryl hydrocarbon receptor-mediated pathway in mouse liver. Toxicol Appl Pharmacol.

[176] Pelclova D, Fenclova Z, Preiss J, Prochazka B, Spacil J, Dubska Z, Okrouhlik B, Lukas E, Urban P (2002). Lipid metabolism and neuropsychological follow-up study of workers exposed to 2,3,7,8- tetrachlordibenzo- p-dioxin. Int Arch Occup Environ Health.

[177] Iwano S, Nukaya M, Saito T, Asanuma F, Kamataki T (2005). A possible mechanism for atherosclerosis induced by polycyclic aromatic hydrocarbons. Biochem Biophys Res Commun.

[178] Curfs DM, Knaapen AM, Pachen DM, Gijbels MJ, Lutgens E, Smook ML, Kockx MM, Daemen MJ, van Schooten FJ (2005). Polycyclic aromatic hydrocarbons induce an inflammatory atherosclerotic plaque phenotype irrespective of their DNA binding properties. FASEB J.

[179] Knaapen AM, Curfs DM, Pachen DM, Gottschalk RW, de Winther MP, Daemen MJ, Van Schooten FJ (2007). The environmental carcinogen benzo[a]pyrene induces expression of monocyte-chemoattractant protein-1 in vascular tissue: a possible role in atherogenesis. Mutat Res.

[180] Pulliero A, Godschalk R, Andreassi MG, Curfs D, Van Schooten FJ, Izzotti A (2015). Environmental carcinogens and mutational pathways in atherosclerosis. Int J Hyg Environ Health.

[181] Penn A (1990). International Commission for Protection against Environmental Mutagens and Carcinogens. ICPEMC working paper 7/1/1. Mutational events in the etiology of arteriosclerotic plaques. Mutat Res.

[182] Zhang YJ, Weksler BB, Wang L, Schwartz J, Santella RM (1998). Immunohistochemical detection of polycyclic aromatic hydrocarbon-DNA damage in human blood vessels of smokers and non-smokers. Atherosclerosis.

[183] Ou X, Ramos KS (1992). Proliferative responses of quail aortic smooth muscle cells to benzo[a]pyrene: implications in PAH-induced atherogenesis. Toxicology.

[184] Ramos KS, Parrish AR (1995). Growth-related signaling as a target of toxic insult in vascular smooth muscle cells: implications in atherogenesis. Life Sci.

[185] Ramos KS, Zhang Y, Sadhu DN, Chapkin RS (1996). The induction of proliferative vascular smooth muscle cell phenotypes by benzo(a)pyrene is characterized by up-regulation of inositol phospholipid metabolism and c-Ha-ras gene expression. Arch Biochem Biophys.

[186] Hough JL, Baird MB, Sfeir GT, Pacini CS, Darrow D, Wheelock C (1993). Benzo(a)pyrene enhances atherosclerosis in white Carneau and show racer pigeons. Arterioscler Thromb.

[187] Curfs DMJ, Lutgens E, Gijbels MJJ, Kockx MM, Daemen MJAP, van Schooten FJ (2004). Chronic exposure to the carcinogenic compound benzo[a]pyrene induces larger and phenotypically different atherosclerotic plaques in ApoE-knockout mice. Am J Pathol.

[188] Thirman MJ, Albrecht JH, Krueger MA, Erickson RR, Cherwitz DL, Park SS, Gelboin HV, Holtzman JL (1994). Induction of cytochrome CYPIA1 and formation of toxic metabolites of benzo[a]pyrene by rat aorta: a possible role in atherogenesis. Proc Natl Acad Sci U S A.

[189] Lehmann AD, Blank F, Baum O, Gehr P, Rothen-Rutishauser BM (2009). Diesel exhaust particles modulate the tight junction protein occludin in lung cells in vitro. Part Fibre Toxicol.

[190] Oesterling E, Toborek M, Hennig B (2008). Benzo[a]pyrene induces intercellular adhesion molecule-1 through a caveolae and aryl hydrocarbon receptor mediated pathway. Toxicol Appl Pharmacol.

[191] Kelly FJ, Fussell JC (2015). Linking ambient particulate matter pollution effects with oxidative biology and immune responses. Ann N Y Acad Sci.

[192] Barath S, Mills NL, Lundback M, Tornqvist H, Lucking AJ, Langrish JP, Soderberg S, Boman C, Westerholm R, Londahl J (2010). Impaired vascular function after exposure to diesel exhaust generated at urban transient running conditions. Part Fibre Toxicol.

[193] Mayati A, Podechard N, Rineau M, Sparfel L, Lagadic-Gossmann D, Fardel O, Ferrec EL (2017). Benzo(a)pyrene triggers desensitization of beta2-adrenergic pathway. Sci Rep.

[194] Alberelli MA, De Candia E (2014). Functional role of protease activated receptors in vascular biology. Vasc Pharmacol.

[195] Brinchmann BC, Le Ferrec E, Podechard N, Lagadic-Gossmann D, Holme JA, Ovrevik J (2018). Organic chemicals from diesel exhaust particles affects intracellular calcium, inflammation and beta-adrenoceptors in endothelial cells. Toxicol Lett.

[196] Kumagai Y, Hayashi T, Miyauchi T, Endo A, Iguchi A, Kiriya-Sakai M, Sakai S, Yuki K, Kikushima M, Shimojo N (2001). Phenanthraquinone inhibits eNOS activity and suppresses vasorelaxation. Am J Physiol Regul Integr Comp Physiol.

[197] Wheeler JL, Martin KC, Resseguie E, Lawrence BP (2014). Differential consequences of two distinct AhR ligands on innate and adaptive immune responses to influenza A virus. Toxicol Sci.

[198] Eckers A, Jakob S, Heiss C, Haarmann-Stemmann T, Goy C, Brinkmann V, Cortese-Krott MM, Sansone R, Esser C, Ale-Agha N (2016). The aryl hydrocarbon receptor promotes aging phenotypes across species. Sci Rep.

[199] Anazawa T, Dimayuga PC, Li H, Tani S, Bradfield J, Chyu KY, Kaul S, Shah PK, Cercek B (2004). Effect of exposure to cigarette smoke on carotid artery intimal thickening: the role of inducible NO synthase. Arterioscler Thromb Vasc Biol.

[200] Brandes RP, Fleming I, Busse R (2005). Endothelial aging. Cardiovasc Res.

[201] Chang CC, Hsu YH, Chou HC, Lee YG, Juan SH (2017). 3-Methylcholanthrene/aryl-hydrocarbon receptor-mediated hypertension through eNOS inactivation. J Cell Physiol.

[202] Gentner NJ, Weber LP (2011). Intranasal benzo[a]pyrene alters circadian blood pressure patterns and causes lung inflammation in rats. Arch Toxicol.

[203] Prins PA, Perati PR, Kon V, Guo Z, Ramesh A, Linton MF, Fazio S, Sampson UK (2012). Benzo[a]pyrene potentiates the pathogenesis of abdominal aortic aneurysms in apolipoprotein E knockout mice. Cell Physiol Biochem.

[204] Ichihara S, Yamada Y, Gonzalez FJ, Nakajima T, Murohara T, Ichihara G (2009). Inhibition of ischemia-induced angiogenesis by benzo[a]pyrene in a manner dependent on the aryl hydrocarbon receptor. Biochem Biophys Res Commun.

[205] Podechard N, Le Ferrec E, Rebillard A, Fardel O, Lecureur V (2009). NPC1 repression contributes to lipid accumulation in human macrophages exposed to environmental aryl hydrocarbons. Cardiovasc Res.

[206] Rigamonti E, Helin L, Lestavel S, Mutka AL, Lepore M, Fontaine C, Bouhlel MA, Bultel S, Fruchart JC, Ikonen E (2005). Liver X receptor activation controls intracellular cholesterol trafficking and esterification in human macrophages. Circ Res.

[207] Jules GE, Pratap S, Ramesh A, Hood DB (2012). In utero exposure to benzo(a)pyrene predisposes offspring to cardiovascular dysfunction in later-life. Toxicology.

[208] Zhang H, Yao Y, Chen Y, Yue C, Chen J, Tong J, Jiang Y, Chen T (2016). Crosstalk between AhR and wnt/beta-catenin signal pathways in the cardiac developmental toxicity of PM2.5 in zebrafish embryos. Toxicology.

[209] Yue C, Ji C, Zhang H, Zhang LW, Tong J, Jiang Y, Chen T (2017). Protective effects of folic acid on PM2.5-induced cardiac developmental toxicity in zebrafish embryos by targeting AhR and Wnt/beta-catenin signal pathways. Environ Toxicol.

[210] Massarsky A, Prasad GL, Di Giulio RT (2018). Total particulate matter from cigarette smoke disrupts vascular development in zebrafish brain (Danio rerio). Toxicol Appl Pharmacol.

[211] Zhang Y, Wang C, Huang L, Chen R, Chen Y, Zuo Z (2012). Low-level pyrene exposure causes cardiac toxicity in zebrafish (Danio rerio) embryos. Aquat Toxicol.

[212] Zhang Y, Huang L, Wang C, Gao D, Zuo Z (2013). Phenanthrene exposure produces cardiac defects during embryo development of zebrafish (Danio rerio) through activation of MMP-9. Chemosphere.

[213] Duan J, Yu Y, Li Y, Wang Y, Sun Z (2016). Inflammatory response and blood hypercoagulable state induced by low level co-exposure with silica nanoparticles and benzo[a]pyrene in zebrafish (Danio rerio) embryos. Chemosphere.

